# Multi-objective sizing and performance optimization of islanded hybrid renewable microgrids: a case study in yanbu, Saudi Arabia

**DOI:** 10.1038/s41598-026-47028-1

**Published:** 2026-04-18

**Authors:** Ayat Ali Saleh, Gaber Magdy

**Affiliations:** 1 Department of Electrical Engineering, Faculty of Energy Engineering, Aswan University, Aswan, 81528 Egypt; 2 Faculty of Engineering, King Salman International University, El-Tor, 46511 Egypt

**Keywords:** Energy management systems (EMS), Hybrid Microgrid System (HMS), Multi-objective Salp Swarm Algorithm (MOSSA), Multi-objective Whale Optimization Algorithm (MOWOA), Loss of Power Supply Probability (LPSP), Cost of Electricity (COE), Renewable factor (RF), Energy science and technology, Engineering

## Abstract

Hybrid microgrid systems (HMS) integrating renewable energy sources (RESs) offer a sustainable solution for residential electrification in remote and arid regions. However, optimal sizing remains challenging due to the trade-offs between reliability, cost, and renewable penetration. This study proposes a multi-objective optimization framework for the techno-economic design of a HMS composed of solar photovoltaic (PV), wind turbine (WT), diesel generator (DG), and battery energy storage system (BESS) units for residential communities in Yanbu, Saudi Arabia. The system performance is evaluated under three load scenarios corresponding to 5, 10, and 15 houses. The optimization problem is formulated to minimize the loss of power supply probability (LPSP) and cost of energy (COE), while maximizing the renewable fraction (RF). Two advanced metaheuristic algorithms, Multi-Objective Salp Swarm Algorithm (MOSSA) and Multi-Objective Whale Optimization Algorithm (MOWOA), are employed and comparatively assessed. A rule-based energy management strategy is integrated to ensure reliable system operation. Results demonstrate that MOSSA provides broader Pareto front coverage and superior solution diversity, while MOWOA achieves competitive cost values in specific scenarios. For the light-load case (5 houses), the optimal PV/WT/BES/DG configuration using MOSSA achieves a COE of 0.45683 $/kWh with an LPSP of 2.0537%, ensuring high reliability. Under normal loading (10 houses), the optimal PV/WT/BES configuration using MOSSA yields a minimum COE of 0.16496 $/kWh, while the DG-integrated configuration reduces LPSP to 16.748% at a COE of 0.26128 $/kWh. For heavy loading (15 houses), the optimized PV/WT/BES/DG system achieves a COE of 0.20873 $/kWh with RF exceeding 88%, demonstrating scalability and strong renewable penetration. Increasing the number of houses leads to higher renewable penetration and reduced dependence on diesel generation. Overall, the proposed framework delivers a scalable and practically applicable solution for residential microgrid deployment in arid regions.

## Introduction

Energy efficiency and conservation are essential to civilization’s ongoing progress. Energy underpins a wide range of essential activities, including residential heating, cooking, lighting, communication systems, contemporary transportation, industrial operations, and healthcare services. The availability of energy is crucial not only for improving the quality of life but also for supporting economic growth. In addition, energy systems play a central role in enabling technological innovation that could potentially address pressing global concerns such as environmental degradation, poverty, and food insecurity^1^. Modern society is fundamentally dependent on energy, which is primarily supplied through two categories of systems: conventional energy systems based on fossil fuels and renewable energy systems (RES). Growing concerns over global warming, climate change, and the depletion of fossil fuels have raised limitations on conventional energy systems. In contrast, RESs offer a sustainable and environmentally friendly alternative, contributing to reduced greenhouse gas emissions and long-term energy security^2^. Wind, hydropower, geothermal, solar, and biomass are among the various types of RES. Among these, wind and solar energy have seen the most widespread development and utilization, owing to their broad availability and substantial environmental benefits. Recently, the expansion of these two RESs has been supported by several factors, including low maintenance costs, ease of installation, growing environmental awareness, and the complementarity with traditional energy systems. Despite the high initial costs for solar and wind energy facilities, they have been widely constructed worldwide due to their ability to minimize shortages in load demand without creating gaps in carbon emissions^3–5^.

Despite significant advancements in renewable energy technologies, many rural villages and islands continue to face electricity shortages. According to the United Nations Development Program (UNDP), around 8% of the world’s population lacks access to electricity, with most of them in rural areas. Extending transmission lines to such regions is often technically infeasible or economically prohibitive due to challenging geographical conditions, including mountainous terrain and dense forests. Suitable electric resources for these areas are RESs such as hydropower, wind, and solar. These resources are typically abundant, clean, and locally available, and their deployment can significantly enhance energy access while promoting energy sustainability in remote regions^6^. Despite these advantages, Countries such as Malaysia are adopting wind power technology very slowly due to low wind speed and the absence of thorough wind assessment, which are key issues. In this context, standalone hybrid or off-grid renewable energy systems have emerged as a promising alternative. These systems integrate multiple RESs to enhance reliability that cannot be guaranteed with a single RES. Hybrid configuration that combine wind turbine (WT), photovoltaic (PV), battery energy storage (BES), supercapacitors (SC), and fuel cell (FC) are particularly efficient because they can reduce output power fluctuations and generate more consistent power. Since standalone systems can be used for isolated and islanded power generation, they seem promising in the near future. Since the hybrid power system is economical and effective, it may be used for different applications in many rural towns and remote villages, such as small-scale lighting and road signage. Since the hybrid power systems must be sized to serve the load with consistent, dependable electricity while also being economical, optimization is crucial^7^.

The most accessible and vital RESs are solar and wind power. When combined, they form a hybrid energy system that offers higher reliability and improved power quality compared to standalone operation. Actually, hybrid energy systems depend essentially on solar and wind power generation; however, their efficiency and robustness can be further enhanced through the integration of auxiliary components such as FCs, diesel generators (DGs), BES, and other energy storage technologies. In fact, these storage devices become essential during periods of low wind speed or solar radiation, as well as during sudden increases in demand^8,9^. Hybrid microgrid system (HMS) architectures represent one of the most reliable, cost-effective, and economical approaches to employ localized RESs. By combining RESs with dispatchable generators, HMS enables localized and concentrated power generation tailored to community needs. These systems come in a variety of scales, from a 5-kW single-phase system to a large three-phase network that provides the neighborhood’s main power source. Moreover, HMSs offer high scalability, allowing seamless expansion and interconnection with the national grid as energy demand grows or the community develops^10^.

Microgrid systems powered by autonomous RESs represent one of the most economical and practical solutions for electrifying off-grid and remote areas. However, the planning and optimal design of such a system are inherently complex from both economic and technological perspectives. A primary challenge arises from the intermittency and weather-dependent nature of RES. To meet the electricity demand, microgrid systems are often either too large or too small. In addition to being expensive to operate, a larger microgrid system will use more energy. On the other hand, a microgrid system that is too small will not be able to supply loads with the electricity they require. Therefore, to fully realize the benefits of RES-based microgrids, effective energy management strategies and optimal microgrid sizing are essential to ensure a reliable, efficient, and cost-effective power supply^11,12^. However, evaluating both the cost and reliability of the system is crucial when optimizing both the size and capacity of the HMS’s components. The two most promising energy sources in the Kingdom of Saudi Arabia, WT and PV systems, are widely deployed, particularly in coastal regions. Because of the incredibly abundant solar resources in the Kingdom, research has been conducted on wind and solar energy since 1985. Several feasibility studies and research have been conducted about the installation of wind energy systems in various areas around the Kingdom. As a result, investments in solar energy projects have expanded significantly in recent years^13,14^.

HMSs have been extensively investigated and discussed in the literature. Although the concept of a hybrid energy system is well established, it has received renewed attention in recent years due to advancements in optimization techniques and energy storage technologies. In^15^, a PV/battery/diesel hybrid system was deployed on Con Dao Island, Vietnam, for minimizing the cost of energy (COE) using the HOMER software. To optimize the annual system cost (ASC) objective function, the Bat Optimization (BO) algorithm has been used in Saudi Arabia for the optimal size of an autonomous PV/WT/BSS/DG hybrid system^16^. Similarly, in^17^, the parallel multi-objective particle swarm optimization (PMOPSO) method was applied to microgrid system sizing problems to find the optimal system configuration of PV, BES, and DGs in Saudi Arabia, considering COE and loss of power supply probability (LPSP) as multi-objective functions. To optimize both COE and LPSP, the multi-objective evolutionary algorithm (MOEA) was used in^18^ to determine the optimal configuration of a microgrid system in Saudi Arabia that includes a DG, PV, and a WT. Its foundation is LPSP enhancement and COE minimization; two goals, COE and LPSP, have been taken into consideration when optimizing a hybrid power generating system that consists of DGs, PV systems, WT units, and BES using the Crow Search Algorithm (CSA)^19^. The optimization problem of the HMS composed of PV systems and BES was addressed in^20^ by minimizing COE and LPSP using various metaheuristic techniques, including Equilibrium Optimizer (EO), Bat Algorithm (BAT), and Black Hole-Based (BHB) methods, which were evaluated in the Egypt-El Dobaa region^20^.

In^21^, a multi-objective problem formulation was proposed using the Multi-Objective Grasshopper Optimization Algorithm (MOGOA) to deal with the optimization problem of hybrid energy systems, with COE and LPSP evaluated for a case study in Nigeria. Similarly, Reference^22^ introduced the social spider optimizer (SSO) technique to optimally size a hybrid renewable energy system deployed in a remote location, incorporating batteries, DG, WT, and PV panels to minimize COE while improving system reliability through LPSP reduction. To meet load demand in Shlateen, Egypt, a HMS consisting of wind, PV, battery, and DG was installed in^23^ with two planned scenarios: PV/wind/battery and PV/wind/battery/diesel. A multi-objective Dragonfly Algorithm (MODA) was applied to solve a multi-objective optimization issue considering three objectives, namely COE, LPSP, and renewable fraction (RF). In^24^, a multi-objective Grey Wolf Optimizer (MOGWO) technique has been utilized to optimize a HMS including solar, WT, diesel, and battery in China. The authors in^25–27^ have suggested the HOMER system to optimize the HMS, considering the minimization of COE and LPSP. It has been tested on several selected stations in Saudi Arabia, India, and Denmark, respectively.

The authors of^28^ suggested a multi-objective problem formulation to perform the optimization challenge using the Simulated Annealing Algorithm (SAA), where the LPSP for the PV/wind/battery HMS has been analyzed. In order to design a HMS as efficiently as possible, another study proposed multi-objective evolutionary algorithms such as Harmony Search (HS), HOMER, Ant Colony Optimization (ACO), and the Jaya algorithm^29^. In^30^, a microgrid system integrating solar PV, wind, DG, and BES were simulated for a case study in Sonderborg, Denmark, where several optimization algorithms have been employed and contrasted to determine the optimal system configuration. Overall, hybrid renewable energy systems have been extensively investigated using a wide range of optimization techniques, as summarized in Table 1.

**Table 1:** Summary of methods applied for optimization and enhancement of hybrid renewable energy systems.

Ref.	Year	Technique/Tool	Case study (Location)	Microgrid System				Objective Function		
				PV	WT	BES	DG	COE	LPSP	RF
^1^	2024	ISSA	isolated microgrid	✓	✓	✓	✓	✓	✓	
^3^	2023	IMOEAD	Yanbu, Saudi Arabia	✓	✓	✓	✓	✓	✓	
^6^	2022	MOSSA	Djelfa, Algeria	✓	✓	✓	✓	✓	✓	
^14^	2021	MONSGA	Saudi Arabia	✓	✓	✓	✓	✓	✓	✓
^15^	2021	HOMER	Con Dao island-Vietnam	✓		✓	✓	✓	✓	
^16^	2022	BO	Saudi Arabia	✓	✓	✓	✓	ASC		
^17^	2021	PMOPSO	Saudi Arabia	✓		✓	✓	✓	✓	
^18^	2021	MOEA	Saudi Arabia	✓	✓		✓	✓	✓	✓
^19^	2022	CSA	Saudi Arabia	✓	✓	✓	✓	✓	✓	
^20^	2022	EQ, BAT, and BHB	Egypt-El Dobaa region	✓		✓		✓	✓	
^21^	2020	MOGOA	Nigeria	✓	✓	✓	✓	✓	✓	
^22^	2020	SSO	Saudi Arabia	✓	✓	✓	✓	✓	✓	
^23^	2019	MODA	Egypt	✓	✓	✓	✓	✓	✓	✓
^24^	2020	MOGWO	China	✓	✓	✓	✓	✓	✓	
^25^	2022	HOMER	Saudi Arabia	✓				✓		
^26^	2021	HOMER	India	✓	✓	✓	✓	✓		
^27^	2022	HOMER	Aalborg, Denmark	✓	✓	✓		✓		
^28^	2021	SAA		✓	✓	✓		✓		
^29^	2022	HS, HOMER, ACO, and Jaya	Yalova, Turkey	✓	✓	✓	✓	ACS		
^30^	2023	MOMFO, NSGA-II, MOPSO, and MOSEO	Sonderborg, Denmark	✓	✓	✓	✓	✓	✓	✓
^31^	2023	HPSODE-FAM, ABC, GA, PSO, and DE	Jiuduansha, China	✓	✓	✓	✓	✓	✓	
^32^	2022	NSGA-II	Tunisia	✓	✓	✓	✓	✓	✓	
^33^	2025	WCA	Yanbu, Saudi Arabia	✓	✓	✓	✓	✓	✓	✓
^34^	2023	PSO	Oujda and Ouarzazate), Spain (Granada), and Algeria (Bechar).	✓	✓	✓	✓	✓		
^35^	2025	HOMER	Hobyo Seaport, Somalia	✓	✓	✓	✓	✓	✓	✓
This study		MOSSA, MOWOA	Yanbu, Saudi Arabia	✓	✓	✓	✓	✓	✓	✓

Recent advancements in hybrid renewable microgrid optimization have increasingly focused on integrating advanced metaheuristic algorithms and multi-dimensional evaluation frameworks. For instance, wind resource assessment combined with hybrid microgrid optimization in low-wind regions demonstrated that coordinated PV, WT, and BESS configurations can significantly reduce the levelized COE while maintaining high reliability levels^36^. Similarly, multi-objective self-adaptive differential evolution techniques have been successfully employed for optimal sizing of PV/wind/diesel systems to simultaneously minimize cost and LPSP^37^. Other studies have explored enhanced swarm-based optimization strategies, such as improved particle swarm optimization for electric vehicle-integrated energy storage systems, achieving considerable operational cost reductions^38^. Moreover, newly developed algorithms, including the Improved Red-Tailed Hawk Algorithm (IRTHA)^39^, Levy-based Salp Swarm Algorithm (LSC-SSA) integrated with supervisory energy management^40^, and advanced bio-inspired optimizers such as BSLO and OOA^41^ have demonstrated improved convergence characteristics and techno-economic performance in off-grid hybrid systems. In addition, comprehensive review studies addressing grid-connected PV systems with retired electric vehicle batteries emphasize the growing importance of intelligent optimization frameworks and flexible energy management strategies in enhancing sustainability and system resilience^42^. These studies collectively highlight the ongoing evolution of optimization methodologies toward more robust, multi-objective, and reliability-oriented hybrid microgrid planning approaches.

In addition, very recent studies have emphasized integrating socio-techno-economic-environmental (STEE) factors into the design of HMSs for off-grid and rural electrification^43–48^. Multi-objective metaheuristic approaches have been applied to optimize PV, WT, BES, biogas (BG), and DG to simultaneously improve technical reliability (LPSP, excess energy), economic performance (COE, total net present cost), environmental sustainability (carbon emissions), and social benefits (employment, human progress index, land use)^43–46^. Marine Predators Algorithm (MPA), Particle Swarm Optimization (PSO), Salp Swarm Algorithm (SSA), and Genetic Algorithm (GA) have been used for optimal sizing of HMS configurations, showing improvements in system reliability and cost-effectiveness^43–47^. Other studies have examined load-side management and optimal dispatch strategies to enhance energy efficiency and reduce unmet load^47,48^. Moreover, recent research has highlighted the importance of integrating predictive dispatch strategies and domestic/telecommunication load considerations into microgrid design to further improve the TEES performance, reduce COE, and enhance reliability in practical applications^49,50^. Compared to these works, the present study focuses on PV/WT/BES/DG-based microgrids for residential communities in Yanbu, Saudi Arabia, considering multiple load scenarios (5, 10, 15 houses), RF as a design constraint, and comparative evaluation of MOSSA and MOWOA algorithms. This integrated approach ensures balanced techno-economic-reliability performance and scalability in arid coastal environments. In designing HMSs, several technical, economic, environmental, and social (TEES) parameters must be considered^45,48^. Technical factors include unmet load, renewable energy penetration, and energy storage capacity. Economic factors cover COE, annualized cost, and total net present cost. Environmental factors involve greenhouse gas emissions and particulate matter. Social factors can include employment generation, land use, and community development. Considering these factors ensures that the designed microgrid is not only reliable and cost-effective but also sustainable and socially beneficial.

The proposed HMS is designed to supply electricity to residential communities with varying load profiles (5, 10, and 15 houses). The load data for each scenario is derived from standard residential consumption patterns, as recommended in recent literature^46,48^. While advanced load scheduling approaches, such as time-shifted loads or integrated load–source side management, have been shown to improve energy efficiency and reduce COE in HMS^47^, recent studies have also demonstrated that integrating predictive load scheduling and source-side management can significantly enhance system efficiency, reduce unmet load, and optimize component utilization for domestic and telecommunication applications^49^. This study focuses on evaluating microgrid sizing and energy management strategies without active load scheduling. This choice allows a clear assessment of the intrinsic system performance, component sizing, and algorithmic efficiency before integrating additional scheduling mechanisms in future work.

Motivated by the aforementioned analysis, this study investigates the optimal allocation of WT, PV, and DG with BES using the Whale Optimization Algorithm (WOA) and Salp Swarm Algorithm (SSA). The objectives are to maximize the share of RESs while minimizing the LPSP and the COE. To evaluate the performance of the proposed system, three load scenarios, light, normal, and heavy, based on the base load, have been considered. Despite the significant progress in hybrid microgrid optimization, several limitations remain in existing literature. Many previous studies focus either on single-objective formulations or treat the renewable fraction as an optimization objective rather than a performance indicator. In addition, most works evaluate system sizing under a single load profile, which limits the understanding of scalability and performance under varying residential demands. Furthermore, although numerous metaheuristic algorithms have been applied to hybrid microgrid design, comparative assessments between recently developed multi-objective variants under identical operating conditions remain limited, particularly for arid coastal regions such as Yanbu, Saudi Arabia. While recent studies have employed advanced variants of SSA, WOA, and other swarm-based optimizers for hybrid microgrid sizing, many of these works primarily emphasize algorithmic performance benchmarking or single-scenario optimization under fixed demand conditions. In several cases, RF is treated solely as an optimization objective rather than a structured design constraint, and limited attention is given to scalability analysis across varying residential load levels. Furthermore, convergence behavior and Pareto front diversity characteristics are often reported descriptively without systematic analytical interpretation under unified constraint configurations. Therefore, there remains a need for an integrated evaluation framework that combines structured multi-objective formulation, algorithmic behavioral analysis, and scalability assessment within a consistent techno-economic-reliability context. To address these gaps, this study proposes a comprehensive multi-objective optimization framework for the optimal sizing of a PV/WT/DG/BESS-based HMS tailored to residential applications. The novelty of this work lies in three main aspects. First, a structured comparative evaluation between the Multi-Objective Salp Swarm Algorithm (MOSSA) and the Multi-Objective Whale Optimization Algorithm (MOWOA) is conducted under identical design constraints and meteorological conditions. Second, system performance is analyzed across three different residential load scenarios (5, 10, and 15 houses) to investigate scalability and design adaptability. Third, the renewable fraction is incorporated alongside reliability and economic metrics to provide a balanced techno-economic-environmental assessment framework. This integrated approach enables a deeper understanding of algorithm behavior, solution diversity, and system scalability for hybrid microgrid deployment in arid coastal environments.

The main contributions of this study can be summarized as follows:

i. A comprehensive multi-objective optimization framework is developed for the optimal sizing of a PV/WT/BESS/DG-based hybrid microgrid tailored to residential applications in Yanbu, Saudi Arabia.
ii. A structured comparative assessment between the MOSSA and the MOWOA is performed under identical design and environmental conditions.
iii. System scalability is investigated through three residential load scenarios (5, 10, and 15 houses), enabling evaluation of performance adaptability under varying demand levels.
iv. The RF is incorporated as a design constraint alongside reliability (LPSP) and economic (COE) objectives, providing a balanced techno-economic-reliability assessment framework.
v. Two system configurations (PV/WT/BESS and PV/WT/BESS/DG) are comparatively evaluated to analyze the impact of diesel integration on cost and reliability performance.

The remainder of this paper is organized as follows: Sect. 2 presents the mathematical model of the HMS. The proposed EMS procedures are provided in Sect. 3. Section 4 provides a detailed explanation of the optimization problem formulation. Section 5 introduces the mathematical model for the proposed optimization approaches. Section 6 presents and discusses the optimization results of the proposed methods. Finally, the paper contributions and findings are concluded in Sect. 7.

## Modelling and configuration of a hybrid microgrid system

### System configuration

**Fig. 1:**
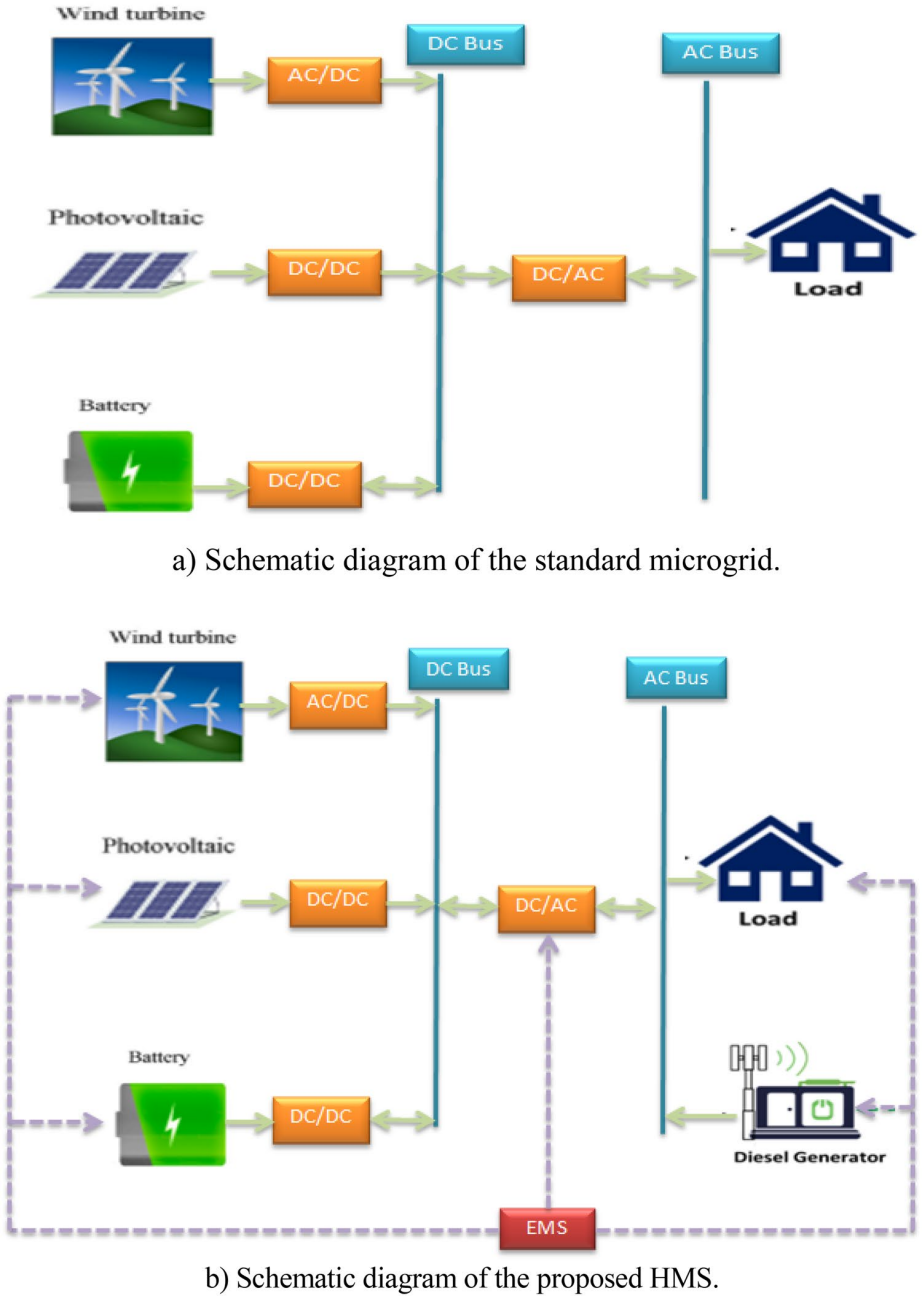
Configuration of standalone microgrid systems.

Figure 1 shows the five main components of the HMS configuration considered in this study. In this setup, the three DC power components are the battery bank, wind plant, and PV plant, while the AC components include the home load and DG.

### A mathematical model of the HMS

To ensure accurate techno-economic evaluation and system reliability assessment, a detailed mathematical modelling framework is developed for each HMS component. The modelling approach is based on hourly time-step simulation over one year, incorporating meteorological data and load demand variations. The generated power from renewable sources, storage dynamics, diesel fuel consumption, inverter efficiency, and system energy balance are mathematically formulated to enable precise performance evaluation and optimization. The following subsections describe the mathematical models of each system component.

#### Photovoltaic system (PVS)

The power produced by a solar PV panel is calculated using Eq. (1), as reported in^51^:

1 $$\:{P}_{pv}\left(\mathrm{t}\right)={N}_{\mathrm{p}\mathrm{v}}\times\:\:\:{A}_{pv}\times\:\:{\mathsf{\eta\:}}_{pv}\times\:\:G\left(t\right)$$

The efficiency of the PV panel is calculated using Eq. (2), as follows:

2 $$\:{\eta\:}_{pv}={\eta\:}_{r}{\eta\:}_{pc}\left[1-{N}_{t}\left(\left({T}_{air}+\left[\frac{{T}_{nom}-20}{800}\right]{R}_{t}\right)-{T}_{ref}\right)\right]$$

#### Wind Energy System (WES)

In a wind power plant, wind energy is captured by a WT and converted into electrical power. The WT’s output at a given location depends primarily on the local wind speed. Since wind speed varies with turbine height, the height of the WT is a critical design parameter. The wind speed as a function of WT height can be computed using the power-law equation as given in Eq. (3), reported in^33^ and^52,53^:

3 $$\:\frac{{v}_{2}}{{v}_{1}}={\left(\frac{{h}_{2}}{{h}_{1}}\right)}^{\alpha\:}$$

As is well known, wind speed has a significant impact on the electricity that wind generators deliver. Theoretically, the power generated by a WT can be evaluated using Eq. (4).

4 $$\:{P}_{wt\_out}\left(t\right)=\left\{\begin{array}{c}0\:\:{\:\:\:\:\:\:\:\:\:\:\:\:\:\:\:\:\:v}_{wind}<{v}_{ci}\:\:\:or\:{v}_{co}\le\:{v}_{wind}\\\:{V}^{3}{\left(\frac{{p}_{r}}{{V}_{rated}^{3}-{V}_{cut\_in}^{3}}\right)-{p}_{r}\left(\frac{{V}_{cu{t}_{in}}^{3}}{{V}_{rated}^{3}-{V}_{cu{t}_{in}}^{3}}\right)\:\:\:\:v}_{ci}\le\:{v}_{wind}<{v}_{r}\\\:{p}_{R}{\:\:\:\:\:\:\:\:\:\:\:\:\:\:\:\:\:\:\:\:\:\:\:\:\:\:\:v}_{r}\le\:{v}_{wind}<{v}_{co}\end{array}\right.$$

#### Diesel generator

To enhance the operational reliability of the HMS, the isolated DG serves as a backup source connected to the AC bus. It plays an essential role in meeting load demands, particularly when RESs are insufficient. As noted in^54^, the efficiency of a DG remains low when operating at low output levels. Therefore, to achieve optimal electricity utilization, the DG must operate at or near its nominal output power. In the event of irregular load demand, this approach offers safe operation against power fluctuations. The generator’s fuel consumption, $\:{\mathrm{q}}_{DG}\left(\mathrm{t}\right)$ is calculated using Eq. (5)^14^, :

5 $$\:{\mathrm{q}}_{DG}\left(\mathrm{t}\right)=\:\mathrm{a}{\mathrm{P}}_{DG}\left(\mathrm{t}\right)+{\upbeta\:}{\mathrm{P}}_{\mathrm{r}\mathrm{a}\mathrm{t}\mathrm{e}\mathrm{d}}$$

where *a* = 0.246 and *b* = 0.08415^55^.

The DG’s efficiency can be computed in Eq. (6), as follows^56^:

6 $$\:{\upeta}_{\mathrm{o}\mathrm{v}\mathrm{e}\mathrm{r}\mathrm{a}\mathrm{l}\mathrm{l}}={\upeta}_{\mathrm{b}\mathrm{r}\mathrm{a}\mathrm{k}\mathrm{e}\:\mathrm{t}\mathrm{h}\mathrm{e}\mathrm{r}\mathrm{m}\mathrm{a}\mathrm{l}}\times\:{{\upeta}}_{\mathrm{g}\mathrm{e}\mathrm{n}\mathrm{e}\mathrm{r}\mathrm{a}\mathrm{t}\mathrm{o}\mathrm{r}}$$

### Inverter

The inverter is an essential operational component for the HMS arrangement taken into consideration in this study. Its primary duty is to convert DC electricity to AC to meet the AC loads’ power needs. The inverter’s efficiency$\:\:\:{\upeta}_{inv}$is approximately calculated as in Eq. (7)^57,58^:

7 $$\:\upeta_{inv}=\frac{P}{P+{P}_{0}+K{P}^{2}}$$

An expression for $\:P$, $\:{P}_{0}$, and $\:K$ are given in Eqs. (11–13), as follows^57–59^:

8 $$\:P=\frac{{P}_{out}}{{P}_{in}}$$

9 $$\:{P}_{0}=1-99{\left(\frac{10}{{\upeta}_{10}}-\frac{1}{{\upeta}_{100}}-9\right)}^{2}$$

10 $$\:K=\frac{1}{{\upeta}_{100}}-{P}_{0}$$

### Battery

In this study, a bank battery is utilized to regulate the power supplied to the load and to store any excess power generated by the system. The battery bank’s state of charge (SOC) is a critical parameter that must be considered in the system operation. The battery operates in two distinct modes, charging and discharging, as described in^60,61^.

A. **Process of charging**:

The charging process of the battery bank can be expressed in Eq. (11), as follows^51^:

11 $$\:{\mathrm{P}}_{\mathrm{c}\mathrm{h}}\left(\mathrm{t}\right)=\left({P}_{PV}\left(t\right)+{P}_{wt}\left(t\right)\right)-\frac{{P}_{load}\left(t\right)}{{\upeta}_{inv}}$$

Furthermore, since hourly iteration time is employed, $\:{E}_{ch}\left(t\right)$ could be used in place of $\:{P}_{ch}\left(t\right)$. The charging mode could be represented mathematically by Eq. (12).

12 $$\:{E}_{batt}\left(t\right)={E}_{batt}\left(t-1\right)+{E}_{ch}\left(t\right)$$

The following condition applies to this expression:

13 $$\:{E}_{ch}\left(t\right)\le\:{E}_{b\:max}-{E}_{b}\left(t\right)$$

B. **Process of discharging**.

The discharge process is expressed in Eq. (14), as follows^51^:

14 $$\:{\mathrm{P}}_{\mathrm{d}\mathrm{c}\mathrm{h}}\left(\mathrm{t}\right)=\frac{{P}_{load}\left(t\right)}{{\upeta}_{inv}}-\left({P}_{PV}\left(t\right)+{P}_{wt}\left(t\right)\right)$$

Furthermore, since hourly iteration time is employed, $\:{E}_{dch}\left(t\right)$ could be used in place of $\:{\mathrm{P}}_{\mathrm{d}\mathrm{c}\mathrm{h}}\left(\mathrm{t}\right)$. The discharging mode could be mathematically presented by Eq. (15).

15 $$\:{E}_{batt}\left(t\right)={E}_{batt}\left(t-1\right)-{E}_{dch}\left(t\right)$$

The following condition applies to this expression:

16 $$\:{E}_{dch}\left(t\right)\le\:{E}_{b}\left(t-1\right)-{E}_{b\:min}$$

### System power balance constraint

At each time step $\:t$, the total generated power must satisfy the load demand and system losses, as expressed by:

17 $$\:{P}_{PV}\left(t\right)+{P}_{WT}\left(t\right)+{P}_{DG}\left(t\right)+{P}_{BESS}\left(t\right)={P}_{Load}\left(t\right)$$

where $\:{P}_{BESS}\left(t\right)$ is positive during discharge and negative during charging. This equation ensures energy equilibrium in the microgrid and is enforced during the optimization process.

As shown in Fig. 1, the energy management strategy ensures coordination between renewable sources, storage, and diesel backup.

## Energy management system of hybrid microgrid system

**Fig. 2:**
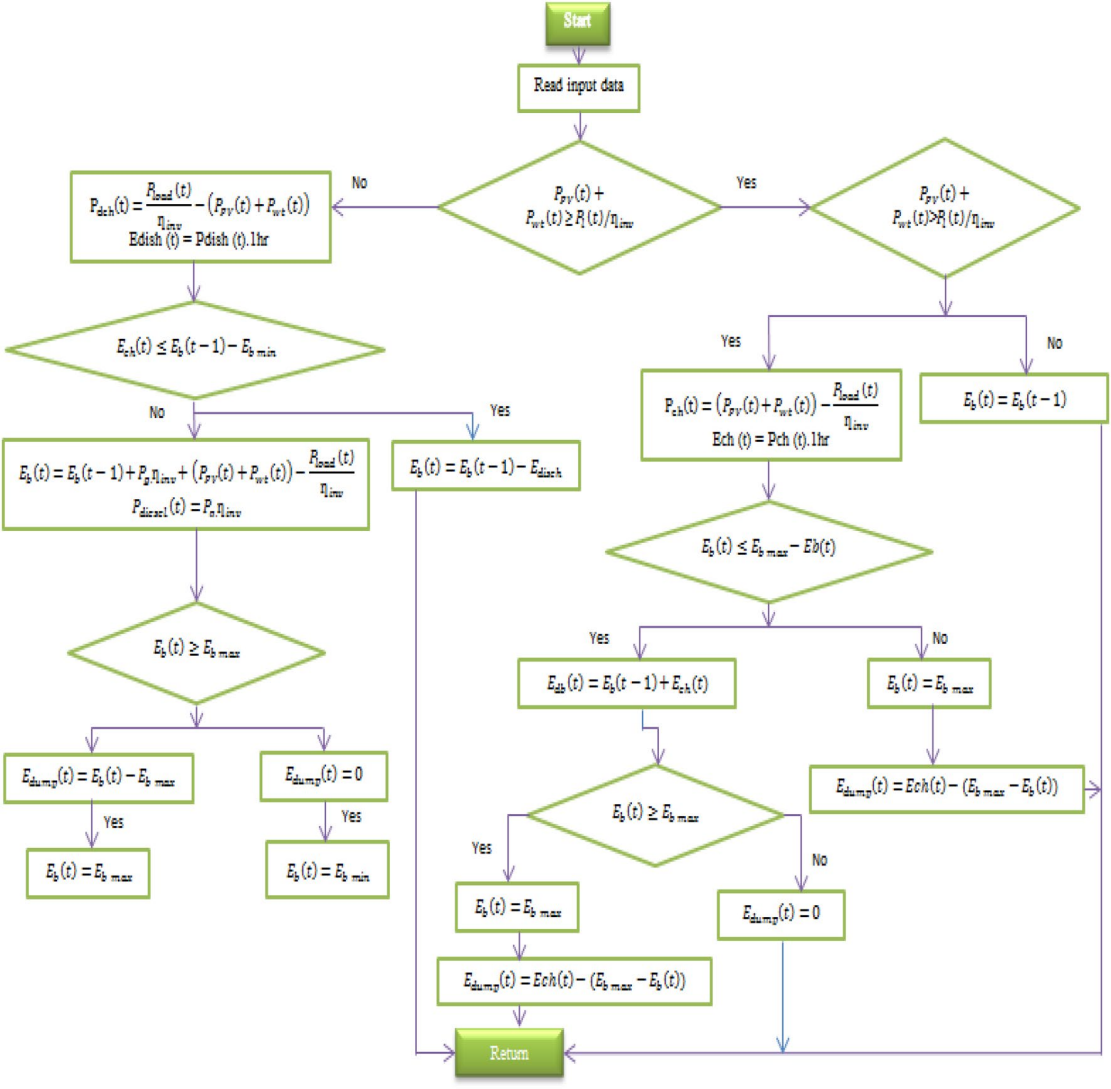
The flowchart of the proposed EMS procedures.

One of the key aspects to be considered when developing or implementing an autonomous microgrid is the EMS. The EMS is responsible for ensuring proper power distribution and control among the various components of the standalone microgrid, thereby enabling optimal power sharing across all HMS components. The flowchart shown in Fig. 2 highlights the management approach adopted by the proposed EMS^1–6^. The main objectives of the proposed EMS are summarized as follows:

- Improving overall efficiency to achieve low-cost and energy-saving operation.
- Maximizing the use of RESs, particularly solar PV and wind power.
- Reducing the fuel consumption of DG.

To achieve these objectives, four EMS operating modes are implemented in this study:

- Mode 1: Priority is given to power generated by RES (WT and PV) to meet the load demand.
- Mode 2: In this mode, after the load demands have been satisfied, any surplus energy produced by RES is used to charge the battery bank.
- Mode 3: When the load demand exceeds the total power generated by the RES, the EMS will attempt to use the battery bank to meet the remaining demand.
- Mode 4: In the worst case, when neither RES nor the battery bank can satisfy the load requirements, the DG is activated to compensate for the power deficit.

## Optimization process

### Problem formulation

This study employs a multi-objective optimization strategy to ensure efficient operation of the HMS. The multi-objective optimization problem considering equality and inequality constraints are given in Eqs. (24–26)^62^:

The formula to reduce is:

18 $$\:\mathrm{F}\left(\mathrm{x}\right)=\left\{{\mathrm{f}}_{1}\left(\mathrm{x}\right),\:{\mathrm{f}}_{2}\left(\mathrm{x}\right),\dots\:,{\mathrm{f}}_{{\mathrm{N}}_{\mathrm{o}\mathrm{b}\mathrm{j}}}\left(\mathrm{x}\right)\right\}$$

Given that:

19 $$\:{\mathrm{g}}_{\mathrm{i}}\left(\mathrm{x}\right)\ge\:0,\:\:\:\:\:\:\mathrm{i}=\mathrm{1,2},\dots\:,{\mathrm{N}}_{\mathrm{u}\mathrm{e}\mathrm{q}}$$

20 $$\:{\mathrm{h}}_{\mathrm{i}}\left(\mathrm{x}\right)\le\:0,\:\:\:\:\:\:i=\mathrm{1,2},\dots\:,{N}_{eq}$$

where x represents a vector of the problem’s design variables. The vector made up of the various objective functions, *f1(x)*,* f2(x)*,*...*,* fk(x).* is denoted by the symbol *F(x).* The equality and inequality constraint sets are represented by the vectors *g(x)* and *h(x)*, respectively.

### Objective function

To guarantee both the cost-effectiveness and reliability of the HMS’s power supply, this study considers three objective functions: RF, COE, and LPSP. Optimal HMS performance is achieved by minimizing the COE and LPSP objective functions while satisfying the associated constraints on the design variables and system operation.

#### Cost of Electricity (COE) of the HMS

A power system’s average cost of producing usable electrical energy during its lifetime is known as the COE, which is frequently used in hybrid energy systems to assess the viability of various asset combinations from an economic standpoint. The net profit cost (NPC) is the principal component in COE analysis and includes capital costs, operation and maintenance expenses, and replacement costs associated with hybrid energy systems. Although RESs generally involve high initial capital costs, they offer several advantages, including high reliability, low operation and maintenance costs, and the absence of fuel expenses. The total cost of the HMS comprises the purchase costs of PV panels, batteries, WT, DG, and inverters. The COE, expressed in $/kWh, can be calculated using Eq. (21)^63,64^.

21 $$\:\mathrm{C}\mathrm{O}\mathrm{E}=\frac{\mathrm{T}\mathrm{o}\mathrm{t}\mathrm{a}\mathrm{l}\:\mathrm{N}\mathrm{e}\mathrm{t}\:\mathrm{P}\mathrm{r}\mathrm{e}\mathrm{s}\mathrm{n}\mathrm{t}\:\mathrm{C}\mathrm{o}\mathrm{s}\mathrm{t}\:\left(\mathrm{T}\mathrm{N}\mathrm{P}\mathrm{C}\right)}{\sum\:_{\mathrm{h}=1}^{\mathrm{h}=8760}{\mathrm{P}}_{\mathrm{l}\mathrm{o}\mathrm{a}\mathrm{d}}}\times\:\mathrm{C}\mathrm{R}\mathrm{F}$$

The capital recovery factor is calculated using Eq. (22).

22 $$\:\mathrm{C}\mathrm{R}\mathrm{F}=\frac{{\mathrm{i}(\mathrm{i}+1)}^{\mathrm{n}}}{{(\mathrm{i}+1)}^{\mathrm{n}}-1}$$

#### Minimization of power loss ($\:\boldsymbol{L}\boldsymbol{P}\boldsymbol{S}\boldsymbol{P}$)

An important statistical method for estimating a system’s reliability power supply is LPSP. It indicates the probability that the demand of the load cannot be satisfied due to insufficient power generation. Power shortfalls may arise from several factors, including economic constraints, uncertainty in RESs availability, and technical failures. The reliability of the system is evaluated using the LPSP, as defined in Eq. (23). The following formula provides the broad definition of LPSP, which is the ratio of energy deficit to total energy demand over a prolonged period of time^7,65^:

23 $$\:\mathrm{L}\mathrm{P}\mathrm{S}\mathrm{P}=\frac{\sum\:{\mathrm{P}}_{\mathrm{L}}\left(\mathrm{t}\right)-({\mathrm{P}}_{\mathrm{W}}\left(\mathrm{t}\right)+{\mathrm{P}}_{\mathrm{P}\mathrm{V}}\left(\mathrm{t}\right)+\left({\mathrm{E}}_{\mathrm{b}}\left(\mathrm{t}-1\right)-{\mathrm{E}}_{\mathrm{b}\mathrm{m}\mathrm{i}\mathrm{n}}\right)+{\mathrm{P}}_{\mathrm{D}\mathrm{G}})}{\sum\:{\mathrm{P}}_{\mathrm{L}}\left(\mathrm{t}\right)}$$

It is important to note that to analyze system reliability, it is assumed that the total load demand exceeds the total power generated as expressed in Eq. (24):

24 $$\:{\mathrm{P}}_{\mathrm{L}}\left(\mathrm{t}\right)>{\mathrm{P}}_{\mathrm{g}\mathrm{e}\mathrm{n}\mathrm{e}\mathrm{r}\mathrm{a}\mathrm{t}\mathrm{i}\mathrm{o}\mathrm{n}}\left(\mathrm{t}\right)$$

#### Renewable factor (RF)

The RF is used to distinguish between the quantity of power generated by renewable and non-renewable sources. Therefore, this factor can be used to quantify the RES’s contribution to the overall power supply. The RF, which reflects the sustainability level of the hybrid system, is computed using Eq. (25). The expression for the RF is as follows^13^:

25 $$\:RF=\left(1-\frac{\sum\:{P}_{diesel}}{\sum\:{P}_{pv}+{P}_{w}}\right)\times\:100$$

**Fig. 3:**
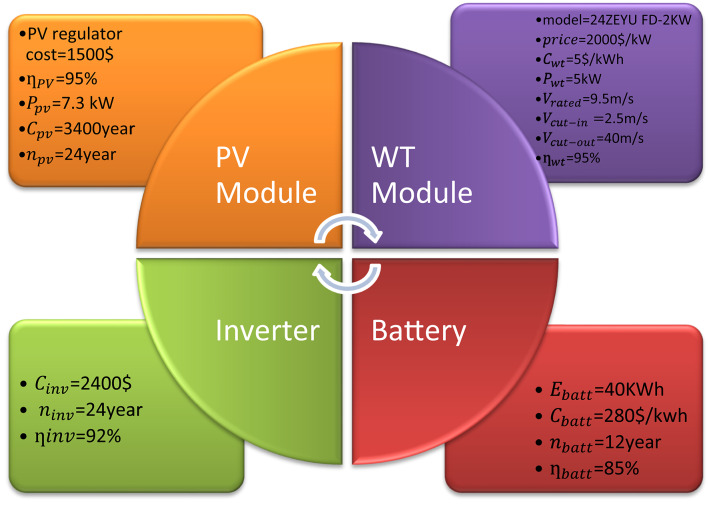
Economic parameters of PV, WT, Battery, and Inverter modules.

It is preferable to have an RF value of 100%, which indicates that the RES meets the entire load demand. Figure 3; Table 1 present the specifications of various generators, batteries, and inverter types used in this study^13^.

**Table 2:** Economic and technical parameters used for HMS.

Component	Parameter	Value	Unit
Diesel generator	$$\:{P}_{DG}$$ $$\:{C}_{DG}$$ $$\:{n}_{DG}$$	4 1000 24,000	Kw $/kW Hours
Economic parameters	$$\:{n}_{project}$$ $$\:fr$$ $$\:{C}_{o\&m}$$ $$\:i$$Discount rate	24 5 20 13 8	Year % % % %

#### Multi-objective pareto formulation

The hybrid microgrid sizing problem is formulated as a multi-objective optimization problem in which the objectives are to minimize the COE and the LPSP, while maximizing the RF. To maintain a consistent minimization framework within the Pareto-based optimization algorithms (MOSSA and MOWOA), the RF is reformulated as a minimization objective by considering (1 − RF). Therefore, the multi-objective problem can be expressed as:

26 $$\:\mathrm{min}F\left(x\right)=[COE\left(x\right),\:LPSP\left(x\right),\:(1-RF\left(x\right)\left)\right]$$

subject to technical and operational constraints of the PV, WT, BESS, and DG units.

The optimization process does not rely on weighted aggregation of objectives. Instead, Pareto dominance principles are employed to identify non-dominated solutions, enabling simultaneous consideration of economic, reliability, and sustainability trade-offs. Solution diversity along the Pareto front is preserved using crowding-distance mechanisms to ensure balanced distribution of optimal design alternatives. This formulation allows decision-makers to select the most appropriate configuration according to practical preferences without imposing predefined objective priorities.

### Constraint

The system operational and design constraints are expressed in Eqs. (27–31). The following constraints define the minimum and maximum allowable sizes of the system components:

- Generation limits:
  27 $$0 \le {N_{pv}} \le N_{pv}^{max}$$
  28 $$\:{0\le\:N}_{wt}\le\:{N}_{wt}^{max}$$
  29 $$\:{0\le\:N}_{Batt}\le\:{N}_{Batt}^{max}$$
- 30 $$\:{0\le\:N}_{DG}\le\:{N}_{DG}^{max}$$
- ♣ Objective limits:

31 $$\:0\le\:LPSP\le\:{LPSP}^{max}$$

$$\:{REF}^{min}\le\:REF$$

## Proposed optimization algorithms

The optimal allocation problem of distributed energy resources (DERs) formulated above constitutes a large-scale, nonconvex, nonlinear optimization problem. Solving such a complex problem requires a robust and effective search strategy. In this context, two well-suited techniques for addressing high-dimensional, complicated issues with improved search quality are the SSA and the WOA.

## Multi-objective salp swarm algorithm

**Fig. 4:**
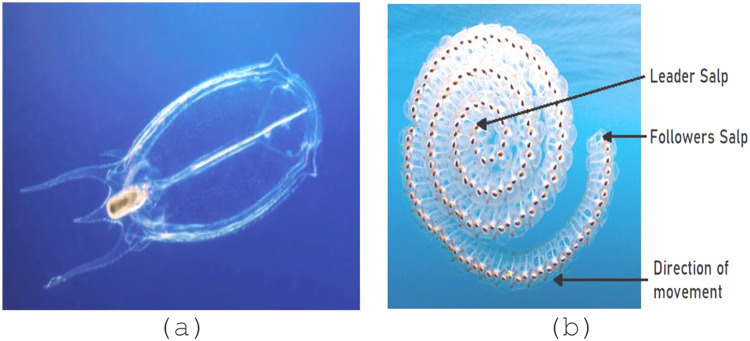
(a) an individual salp, (b) a chain of salps,.

**Fig. 5:**
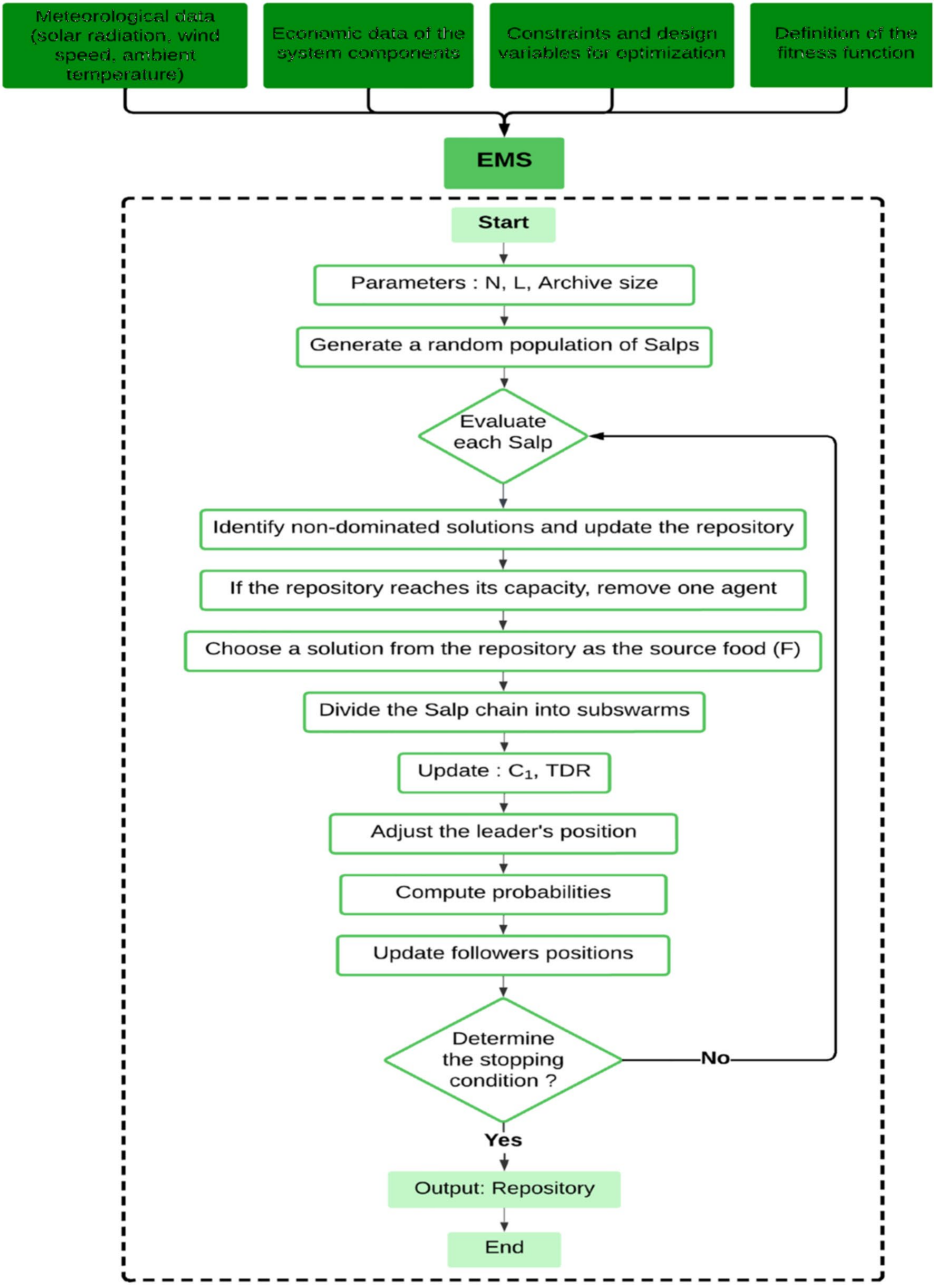
Flowchart of the SSA.

The SSA swarm intelligence algorithm-based metaheuristic optimization technique was proposed by Mirjalili et al. in 2017^66^. It is inspired by the collective intelligence and foraging habits of salps, marine organisms that form chains-like structures while searching for food. To capture the food source, every series will follow the leader (lead salp), and the leader will guide and lead the other salps^67^. Figure 4(a) depicts the structure of a salp, whereas Fig. 4(b) shows the salp chain. The flowchart of the proposed MOSSA approach is shown in Fig. 5.

In the search space, the salp leader approaches the food source designated ($\:{F}_{J}$), as all followers are free to approach the remaining salp. The n-dimensional search space contains the salps’ location, in a particular problem, n represents the number of variables. Accordingly, the position of every salp is kept in a two-dimensional matrix (N*d), denoted by $\:{\mathrm{X}}_{i}$, and is explained by Eq. (32)^68^:

32 $$\:{\mathrm{X}}_{i}=\left[\begin{array}{cccc}{X}_{1}^{1}&\:{X}_{2}^{1}&\:\cdots\:&\:{X}_{d}^{1}\\\:{X}_{1}^{2}&\:{X}_{2}^{2}&\:\cdots\:&\:{X}_{d}^{2}\\\:\vdots&\:\vdots&\:\cdots\:&\:\vdots\\\:\vdots&\:\vdots&\:\cdots\:&\:\vdots\\\:{X}_{1}^{N}&\:{X}_{2}^{N}&\:\cdots\:&\:{X}_{d}^{N}\end{array}\right]$$

The leader’s position is updated as expressed in Eq. (33)^66^:

33 $$\:{\mathrm{x}}_{j}^{1}=\left\{\begin{array}{c}{F}_{J}+{C}_{1}\left(\left(u{b}_{j}-l{b}_{j}\right){C}_{2}+l{b}_{j}\right)\:\:\:\:{C}_{3}\ge\:0\\\:{F}_{J}-{C}_{1}\left(\left(u{b}_{j}-l{b}_{j}\right){C}_{2}+l{b}_{j}\right)\:\:\:\:{C}_{3}<0\end{array}\right.$$

The exploration and exploitation are balanced by the parameter c_1_, Thus, it is regarded as one of the SSA’s most crucial parameters, and its definition provided in Eq. (34)^6,66^:

34 $$\:{\mathrm{C}}_{1}=2{\mathrm{e}}^{-{\left(\frac{\varDelta\:\mathrm{i}}{L}\right)}^{2}}$$

The followers can adjust their places according to Newton’s law of motion given in Eq. (35)^6,66^:

35 $$\:{\mathrm{x}}_{j}^{i}=0.5a{t}^{2}+{v}_{0}t$$

where $$\:\:i\ge\:2$$, $$\:a\:=\:\frac{{v}_{final}}{{v}_{0}}$$ where $$\:v\:=\:\frac{x-{x}_{0}}{t}$$.

Since the optimization time indicates the iteration according to a designated sample unit, given that $\:{v}_{0}$= 0, the difference between iterations is equal to 1, the aforementioned equation has been expressed as follows^6,66^:

36 $$\:{\mathrm{x}}_{j}^{i}=0.5({x}_{j}^{i}+{x}_{j}^{i-1})$$

## Mathematical model of WOA

**Fig. 6:**
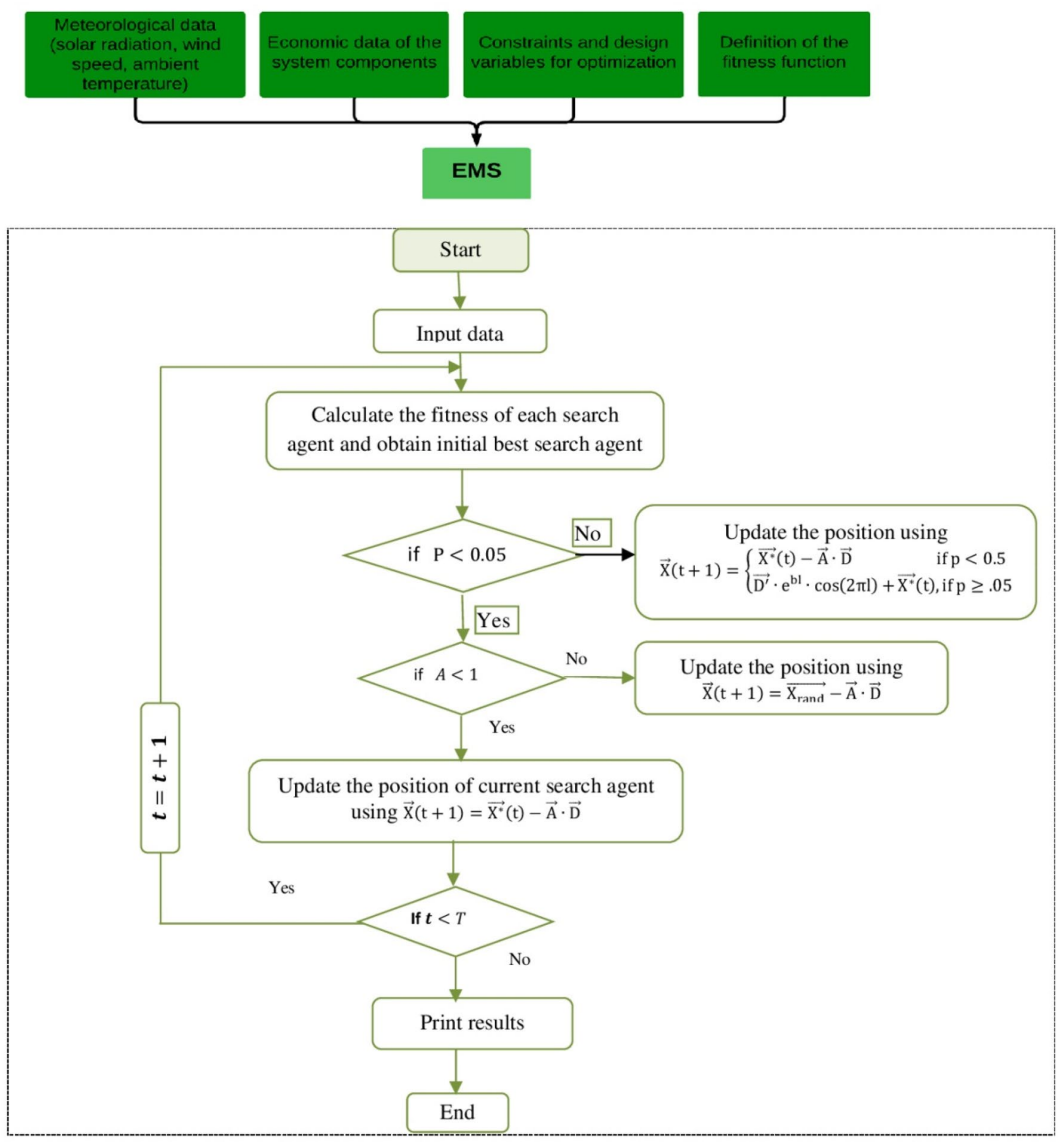
Flowchart of the WOA.

The WOA algorithm is inspired by the bubble-net feeding approach employed by humpback whales during hunting. In this behavior, humpback whales dive to depths of approximately 10–15 m and generate spiral-shaped bubble nets to encircle their prey, after which they follow the bubble path while ascending toward the surface^69^. The flowchart of the WOA approach is illustrated in Fig. 6.

### Encircling prey

When hunting, humpback whales locate their prey and encircle them in the manner described in Eqs. (37–38)^70^.

37 $$\:\overrightarrow{\mathrm{D}}=\left|\overrightarrow{\mathrm{C}}\cdot\:\overrightarrow{{\mathrm{X}}^{\mathrm{*}}}\left(\mathrm{t}\right)-\overrightarrow{\mathrm{X}}\left(\mathrm{t}\right)\right|$$

38 $$\:\overrightarrow{\mathrm{X}}\left(\mathrm{t}+1\right)=\overrightarrow{{\mathrm{X}}^{\mathrm{*}}}\left(\mathrm{t}\right)-\overrightarrow{\mathrm{A}}\cdot\:\overrightarrow{\mathrm{D}}$$

The coefficient vectors A and C could be formulated using Eqs. (39–40):

39 $$\:\overrightarrow{\mathrm{A}}=2\overrightarrow{\mathrm{a}}\cdot\:\overrightarrow{\mathrm{r}}-\overrightarrow{\mathrm{a}}$$

40 $$\:\overrightarrow{\mathrm{C}}=2\cdot\:\overrightarrow{\mathrm{r}}$$

### Bubble-net attacking method

To statistically simulate humpback whale bubble-net activity, two approaches are organized as follows^71^.

- **Shrinking encircling mechanism**: This approach is organized by setting random values for $\:\overrightarrow{\mathrm{A}}$ in [− 1, 1] and minimizing the value of $\:\overrightarrow{\mathrm{a}}$ in Eq. (39).
- **Spiral updating position**: After calculating the distance between the whale and its prey, a spiral equation is created to show the humpback whales’ helix-shaped movement as given in Eq. (41):

41 $$\:\overrightarrow{\mathrm{X}}\left(\mathrm{t}+1\right)=\overrightarrow{{\mathrm{D}}^{{\prime\:}}}\cdot\:{\mathrm{e}}^{\mathrm{b}\mathrm{l}}\cdot\:\mathrm{cos}\left(2{\uppi\:}\mathrm{l}\right)+\overrightarrow{{\mathrm{X}}^{\mathrm{*}}}$$

42 $$\:\overrightarrow{{\mathrm{D}}^{{\prime\:}}}=\left|\overrightarrow{{\mathrm{X}}^{\mathrm{*}}}\left(\mathrm{t}\right)-\overrightarrow{\mathrm{X}}\left(\mathrm{t}\right)\right|$$

A probability of 0.5 is assumed to switch between the spiral updating model and the shrinking encircling mechanism, thereby mimicking the simultaneous hunting behaviors of humpback whales. During the exploitation phase, the positions of whales are updated according to the formulations in Eq. (43):

43 $$\:\overrightarrow{\mathrm{X}}\left(\mathrm{t}+1\right)=\left\{\begin{array}{c}\overrightarrow{{\mathrm{X}}^{\mathrm{*}}}\left(\mathrm{t}\right)-\overrightarrow{\mathrm{A}}\cdot\:\overrightarrow{\mathrm{D}}\:\:\:\:\:\:\:\:\:\:\:\:\:\:\:\:\:\:\:\:\:\:\:\:\:\:\:\:\:if\:p<0.5\\\:\overrightarrow{{\mathrm{D}}^{{\prime\:}}}\cdot\:{\mathrm{e}}^{\mathrm{b}\mathrm{l}}\cdot\:\mathrm{cos}\left(2{\uppi\:}\mathrm{l}\right)+\overrightarrow{{\mathrm{X}}^{\mathrm{*}}}\left(\mathrm{t}\right)\:\:\:\:\:if\:p\ge\:.05\end{array}\right.$$

### Search for prey (exploration phase)

Humpback whales search randomly depending on each other’s circumstances. In this exploration phase, search agents are forced to wander far away from a reference whale because $\:\overrightarrow{\mathrm{A}}$ is supposed to be higher than 1 or less than − 1. In the exploration phase, the position of a randomly selected search agent is given in Eqs. (44–445) when$\:\left|\overrightarrow{\mathrm{A}}\right|>1$.

44 $$\:\overrightarrow{\mathrm{D}}=\left|\overrightarrow{\mathrm{C}}\cdot\:\overrightarrow{{\mathrm{X}}_{\mathrm{r}\mathrm{a}\mathrm{n}\mathrm{d}}}-\overrightarrow{\mathrm{X}}\right|$$

45 $$\:\overrightarrow{\mathrm{X}}\left(\mathrm{t}+1\right)=\overrightarrow{{\mathrm{X}}_{\mathrm{r}\mathrm{a}\mathrm{n}\mathrm{d}}}-\overrightarrow{\mathrm{A}}\cdot\:\overrightarrow{\mathrm{D}}$$

## Parameter space exploration and convergence strategy

To ensure efficient convergence and adequate exploration of the decision space, the optimization process is conducted within predefined technical bounds for all design variables. The lower and upper limits of PV capacity, WT units, battery autonomy days, and DG units were selected based on practical deployment constraints and regional feasibility conditions. In the MOSSA algorithm, the adaptive coefficient $\:{C}_{1}$decreases exponentially with iteration number, allowing broad exploration during early iterations and enhanced exploitation in later stages. Similarly, in MOWOA, the parameter $\:a$ decreases linearly to transition gradually from exploration (|A| > 1) to exploitation (|A| < 1), ensuring balanced search behavior. To avoid premature convergence and maintain solution diversity, Pareto dominance sorting and crowding-distance mechanisms were employed during the multi-objective selection process. This preserves non-dominated solutions and ensures uniform Pareto front distribution. Furthermore, convergence performance is monitored by tracking the evolution of objective functions (COE, LPSP, and RF) across iterations. Stable Pareto fronts and negligible objective improvements in final iterations indicate effective convergence of both algorithms.

In addition, the population size and maximum number of iterations were selected based on preliminary numerical testing to ensure convergence stability without excessive computational burden. Several trial runs were conducted to verify that the chosen parameter settings provide consistent Pareto front convergence. The same parameter configuration was maintained for both MOSSA and MOWOA to ensure fair performance comparison. This parameter control strategy contributes to maintaining an appropriate balance between exploration and exploitation throughout the optimization process.

## Results and Discussion

This paper presents several optimal design alternatives for the microgrid under study using two multi-objective optimization methodologies, namely MOSSA and MOWOA. These methods generate the Pareto front comprising a set of optimal trade-off solutions. Two HMS configurations, PV/WT/BES and PV/WT/BES/DG, are optimized for Yanbu, Saudi Arabia, under three different load scenarios corresponding to communities of five, ten, and fifteen homes.

**Fig. 7:**
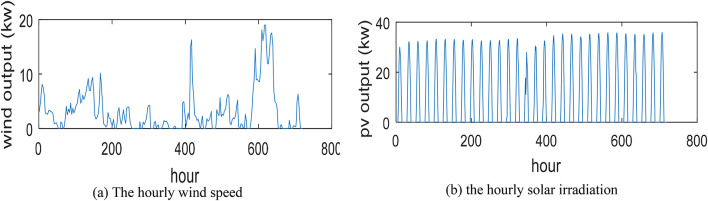
The hourly variations of wind and solar PV power.

Saudi Arabia is characterized by a hot and dry climate with abundant RES, particularly solar and wind. Yanbu, an industrial city located on the Red Sea coast in the western part of the Kingdom (latitude 24°05′20″ N and longitude 38°03′49″ E), experiences high solar irradiation and favorable wind conditions. This study considers the annual average wind speed of 3.53 m/s and an average solar radiation of 5.95 kWh/m^2^/day^13^. The hourly variations of wind speed and solar irradiation in Yanbu are illustrated in Fig. 7. Ambient temperatures in the city range from 15 °C to 40 °C, with an annual average of approximately 29 °C. The optimization process employs real meteorological data for Yanbu. The lower and upper bounds for PV, BES, WT, and DG capacities are specified as^15,45^, [0, 10], and^1,4^, respectively. Table 3 presents six distinct case studies based on three scenarios, considering different numbers of households and microgrid system configurations.

**Table 3:** The different cases studied in this paper.

Scenario	Case	Case studied	Loading (Number of houses)
1	1	PV/WT/BES	light loading (*N* = 5)
	2	PV/WT/BES/DG	
2	3	PV/WT/BES	Normal loading (*N* = 10)
	4	PV/WT/BES/DG	
3	5	PV/WT/BES	Heavy loading (*N* = 15)
	6	PV/WT/BES/DG	

## Scenario 1: Light loading condition

The proposed multi-objective optimization methods are applied to simultaneously minimize the COE and the LPSP, while maximizing the utilization of RESs. This is achieved by optimally allocating different types of energy resources, including RES (PV and WT), BES units, and non-RESs like diesel. In this scenario, the HMS is analyzed under a light load condition with a total of five households.

### A. Case 1

B. **PV/WT/BES Design**.

**Fig. 8:**
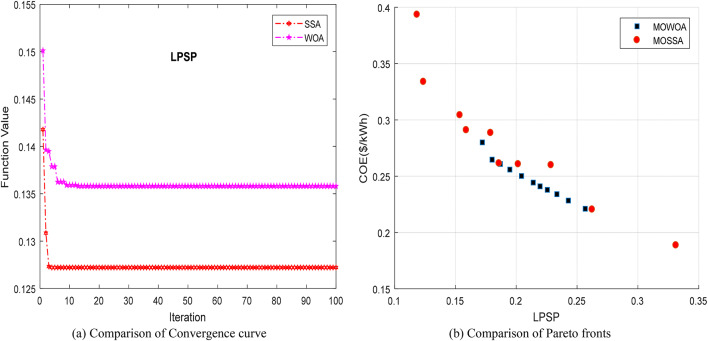
Comparison of Convergence curve and Pareto fronts obtained for Case 1 using the two proposed approaches.

This design uses the three components, PV/WT/BES, that provide the minimum required energy, where PV and WT constitute the RESs and the BES serves as a backup to supply power when other sources are insufficient. The convergence curve of several intelligence algorithms is shown in Fig. 8 (a). After 5 iterations, the SSA achieves a higher convergence accuracy and reaches the optimal value before the competing algorithms. The Pareto front generated by the proposed algorithms, considering the two objective functions (LPSP and COE) for the standalone HMS is illustrated in Fig. 8 (b). It is observed that the Pareto front of MOSSA covers a wider area of decision maker choices compared to the MOWOA, and the solutions of MOSSA are dominated by MOWOA, especially in the mid portion of the Pareto front.

Based on Pareto fronts, both algorithms generate ten optimal solutions, as summarized in Tables 4 and 5. These solutions have been arranged according to the LPSP for greater clarity. Table 4 makes it evident that if the designer chooses option #1, the electricity produced by the PV panels will be 15 kW, the system provides 1.1944 days of autonomy, and six WT are needed. This configuration corresponds to an LPSP of 33.124% and a COE of 0.18872 $/kWh. In contrast, option #10 requires ten WTs, 4.2932 days of autonomy, and a total PV generation of 30.636 kW, resulting in an LPSP of 11.839% and a COE of 0.39358 $/kWh. From Table 5, it is evident that simulations of solution#1 provide the lowest COE of 0.22108 $/kWh using MOWOA method. Among all the solutions, solution #10 achieves the lowest LPSP and the highest contribution of energy from PV panels (20.657 kW), with a corresponding COE of 0.28004 $/kWh. All system constraints are satisfied in this configuration: the BAD is 4.0962 days, and the PV array supplies 20.657 kW of power, and seven WT are required.

**Table 4:** Optimization results for the MOSSA method under Case 1.

Solution	PV (kW)	BAD	WT	COE ($/kWh)	LPSP
1	15	1.1944	6	0.18872	0.33124
2	16.767	3.0328	5	0.22052	0.26234
3	22.737	3.6047	3	0.25995	0.22857
4	15	3.3981	10	0.26077	0.20157
5	17.908	4.5495	6	0.26153	0.1858
6	22.311	3.5931	7	0.28856	0.17889
7	18.238	4.1692	10	0.29101	0.15886
8	22.492	4.0757	8	0.30432	0.15361
9	23.545	4.5883	9	0.33389	0.12369
10	30.636	4.2932	10	0.39358	0.11839

**Table 5:** Optimization results for the MOWOA method under Case 1.

Solution	PV (kW)	BAD	WT	COE ($/kWh)	LPSP
1	15	3.5632	6	0.22108	0.25674
2	15.929	3.3897	6	0.22658	0.24572
3	15.929	3.6624	6	0.23024	0.23906
4	16.019	4.0962	7	0.24355	0.21568
5	16.462	4.1177	6	0.24463	0.21335
6	16.611	4.1327	6	0.2452	0.21237
7	17.066	4.5468	6	0.25591	0.19471
8	17.159	4.8665	6	0.26088	0.18691
9	17.565	4.8739	6	0.26506	0.17985
10	20.657	4.0962	7	0.28004	0.17212

C. **Case 2: PV/WT/BES/DG Design**.

**Fig. 9:**
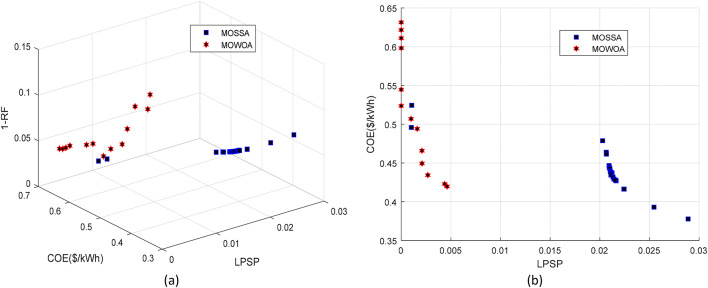
Comparison of Pareto fronts obtained for Case 2 using the two proposed approaches.

The PV/WT/BES/DG framework consists of four components that collectively provide the maximum energy output. Renewable energy is supplied by the PV and WT units, while the BES and DG serve as backup sources to ensure power availability when the primary renewable sources are insufficient. The Pareto front of the standalone microgrid system, obtained using each algorithm and based on LPSP, COE, and RF functions, is shown in Fig. 9. The findings on the Pareto front display a range of design decision options in addition to an ideal solution and a collection of optimal solutions (non-dominated solutions). Compared to the MOWOA, the Pareto front generated by the proposed MOSSA spans a wide range of choices for decision makers. The detailed outcomes of the MOSSA and MOWOA algorithms are presented in the following section.

Based on Pareto fronts, the MOSSA algorithm provides ten optimal solutions, which are listed in Table 6. For clarity, these solutions are arranged according to the LPSP. Solution #1 exhibits the highest LPSP and lowest COE and RE among the obtained solutions, with a COE of 0.28885$/kWh, an LPSP of 15.59%, and an RF of 57.772%). In contrast, Solution #10 achieves the lowest LPSP value, and the most favorable COE and RE values, corresponding to a COE of 0.52238 $/kWh, 0.097% LPSP, and 94.076% RF. For this optimal configuration, the PV system generates 35.208 kW of power, the battery is 4.8093 days, and the system requires 10 WT, and 2 DGs. Based on Pareto fronts, the MOWOA algorithm provides ten solutions, which are listed in Table 7. According to the outcomes, if the designer chooses solution #1, the PV system generates 22.273 kW of power, the battery autonomy days (BAD) period is 4.9917 days, and 10 WT are needed. The corresponding COE, LPSP, and RF values of this solution are 0.37779 $/kWh, 2.8887%, and 93.89%, respectively. This solution exhibits a relatively higher LPSP compared to the other available options, while achieving lower COE and RF values. If the designer chooses solution #10, the PV system generates 36.003 kW of power, the BAD period is five days, and the system requires ten WT and two DGs. For this configuration, the COE, LPSP, and RF are 0.52435 $/kWh, 0.10552%, and 94.384%, respectively. Compared to the other available solutions, this option achieves a significantly lower LPSP at the expense of a higher COE, indicating a trade-off between system reliability and economic cost.

**Table 6:** Optimization Results for the MOSSA method under Case 2.

Solution	PV (kW)	BAD	WT	Diesel	COE ($/kWh)	LPSP	RF
1	15.318	2.761	1	1	0.28885	0.15592	0.57721
2	16.065	2	7	1	0.3038	0.097285	0.83316
3	21.239	2.3991	8	1	0.34432	0.079308	0.88536
4	22.035	4.4168	10	1	0.37061	0.038405	0.933
5	33.062	5	10	1	0.45683	0.020537	0.95712
6	26.68	4.9404	10	2	0.46207	0.0011175	0.92708
7	24.277	4.9374	10	2	0.44564	0.0014363	0.92081
8	20.206	4.8693	10	3	0.49429	0	0.89304
9	30.552	4.7865	10	3	0.5512	0	0.92733
10	35.208	4.8093	10	2	0.52238	0.00097779	0.94076

**Table 7:** Optimization Results for the MOWOA method under Case 2.

Solution	PV (kW)	BAD	WT	Diesel	COE($/kWh)	LPSP	RF
1	22.273	4.9917	10	1	0.37779	0.028887	0.9389
2	24.408	4.9982	10	1	0.39299	0.025454	0.9442
3	27.565	4.9992	10	1	0.4162	0.022448	0.94986
4	29.727	4	10	1	0.43177	0.021322	0.95265
5	30.343	5	10	1	0.43679	0.021232	0.95381
6	30.502	4.9952	10	1	0.43761	0.02118	0.95373
7	31.204	5	10	1	0.44284	0.021004	0.95467
8	33.732	4.9973	10	1	0.46139	0.020663	0.95771
9	34.899	5	10	2	0.51428	0.0011761	0.94131
10	36.003	5	10	2	0.52435	0.0010552	0.94384

D. **A comparative analysis of the contributions of microgrid energy generation**.

**Fig. 10:**
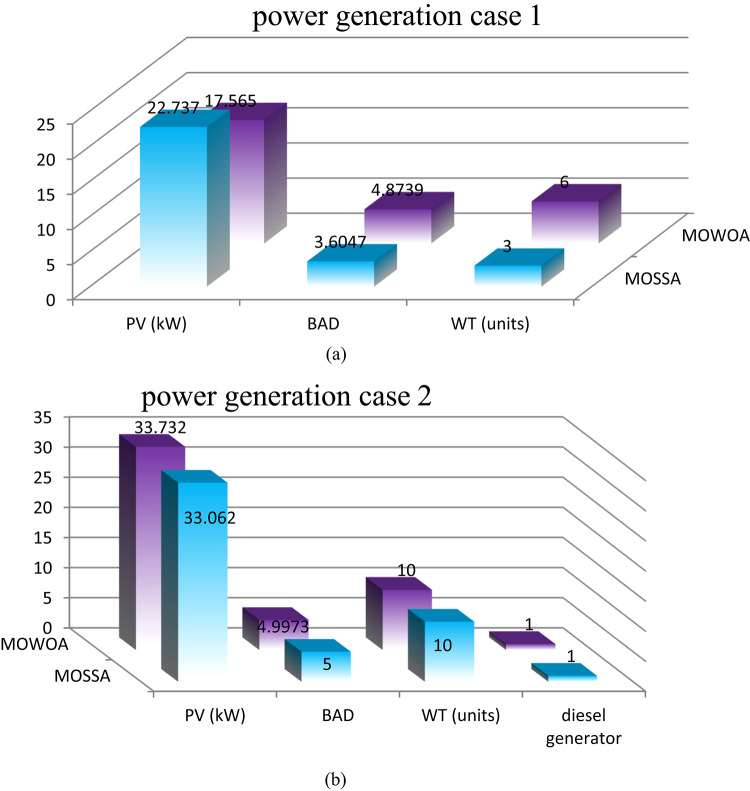
Comparison of the component sizes of proposed architecture under different proposed methods.

Two different design configurations of microgrid components, PV/WT/BES/DG and PV/WT/BES, are examined in this study. The energy contributions are compared for both configurations to evaluate their performance. To solve the optimization problem examined in this research, the performance of the MOSSA method is compared with that of the MOWOA algorithm. The energy contributions of various sources, such as PV system, WT, BES, and DG are contrasted in Fig. 10 (a, b) and Table 8. The results demonstrated that:

- The PV/WT/BES/DG configuration, which comprises four components, provides the highest total energy. The renewable energy is supplied by PV and WT, while the backup power is provided by the BES and DG supply in case of energy shortage.
- With only three components, the PV/WT/BES configuration generates less total energy than the other combination (PV/WT/BES/DG). In this architecture, the BES serves as the sole backup source when renewable energy sources are insufficient.
- The MOSSA algorithm produces a lower total installed PV capacity than the MOWOA method for case 2.
- The MOWOA algorithm tends to allocate a higher number of WT (up to 10 WTs) with a small number of DGs for case 2.
- **For the PV/WT/BES architecture**.
- The average number of WT and the total PV capacity achieved from the MOSSA, are higher than those achieved from the MOWOA, but the average number of BES is less.
- **For the PV/WT/BES/DG architecture**.
- For the MOSSA approach, the power contributed by PV units ranges from 15.318 to 35.208 kW, whereas for the MOWOA method, it ranges from 22.273 to 36.003 kW.
- The MOWOA method obtained WT and BES numbers that are both close to the upper bounds of^5,10^.
- The number of WT s taken from MOWOA ranges from 1 to 10 units.
- For MOWOA and MOSSA techniques, the number of DG falls between^1,2^ and^1–3^, respectively.
- The MOWOA algorithm tends to allocate a higher average number of WT, PV, BAD, and DGs.

**Table 8:** Min, Max and average Limits of Size of different Energy Resources in Scenario 1.

Component	limits	Case 1		Case 2	
		MOSSA	MOWOA	MOSSA	MOWOA
PV panel	Min	15	15	15.318	22.273
	Max	30.636	20.657	35.208	36.003
	Average	20.4634	16.8397	24.464	30.065
BAD	Min	1.1944	3.5632	2	4
	Max	4.2932	4.0962	4.9404	5
	Average	3.6499	4.1345	4.0919	4.8985
WT	Min	3	6	1	10
	Max	10	7	10	10
	Average	7.4	6.2	8.6	10
DG	Min	--	--	1	1
	Max	--	--	3	2
	Average	--	--	1.6	1.1

E. **Comparative analysis of the LPSP**,** COE**,** and RF**.

**Fig. 11:**
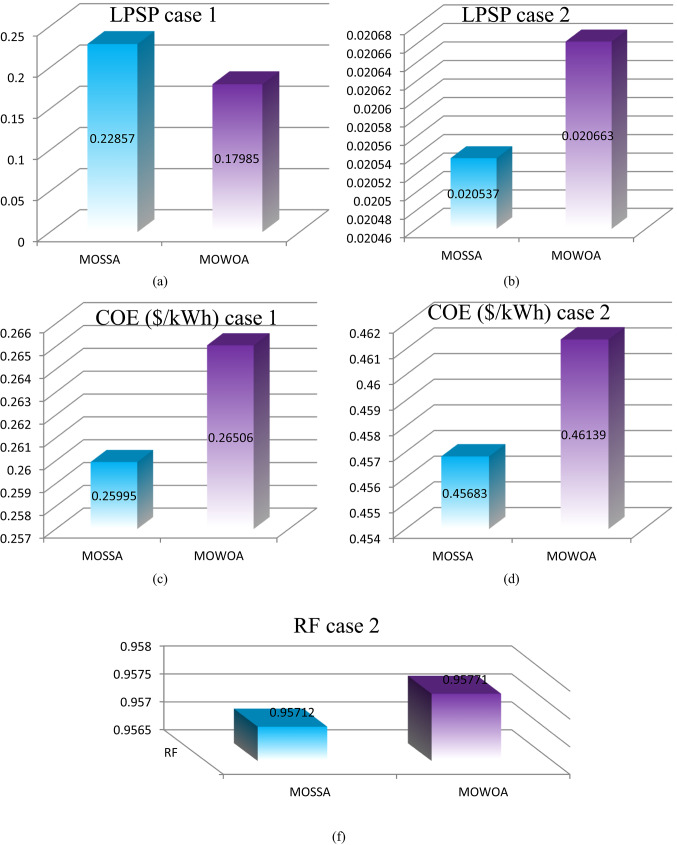
Comparison of LPSP, COE, and RF of the proposed architecture under different proposed methods.

Figure 11 (a, b, c, d, and e) displays the numerical fluctuations of the LPSP, COE, and RF indices in different models for various instances. As can be observed,

- The PV/WT/BES/DG configuration produces more COE and less LPSP than the PV/WT/BES architecture due to having four components.
- All limitations are respected in all cases, with the PV/WT/BES/DG design having an LPSP of less than 15% and the PV/WT/BES design having an LPSP of less than 33%.
- According to the MOWOA algorithms, the RF performed well in all cases, surpassing 83.316%.
- **For the PV/WT/BES/DG architecture**.
- Among all optimal solutions, the MOSSA algorithm provides the best-performing configuration, achieving a COE of 0.45683 $/kWh and an LPSP of 2.0537%.
- The MOWOA algorithm yielded more costly results than the MOSSA, with an LPSP of 20.663 and a COE of 0.46139$/kW h. On the other hand, MOWOA offered the best RE sharing among the previous algorithms.
- **For the PV/WT/BES architecture**.
- Solution #3 is the most appropriate outcome for the case study, according to the conclusions based on the MOSSA technique. The system’s COE in this configuration is 0.25995 $/kWh. The limitations have been met: the system receives 22.737 kW of power from PV, the LPSP is 22.857%, and the day of autonomy for BESU is set at 3.6047.
- According to the findings of the MOWOA methodology, solution # 9 is the most suitable result for the case study. In this configuration, the COE of the system is 0.26506 $/kWh. The system receives 17.565 kW of power from PV, the LPSP is 17.985%, and the day of autonomy for BESU is set at 4.8739. These limitations have all been met.
- Compared to the MOSSA, MOWOA algorithms offered more costly solutions.
- The MOWOA algorithms provided lower loss solutions compared to MOSSA.

## Scenario 2: Normal loading condition

The proposed multi-objective optimization methods are employed to simultaneously minimize the COE and the LPSP while maximizing the utilization of RES. This is achieved through the optimal allocation of various energy resources, including RES (PV and WT), BES, and non-RES like DG. In this scenario, A Microgrid system is operating at normal load conditions, supplying electricity to a community comprising 10 houses.

### A. Case 3

B. **PV/WT/BES Design**.

**Fig. 12:**
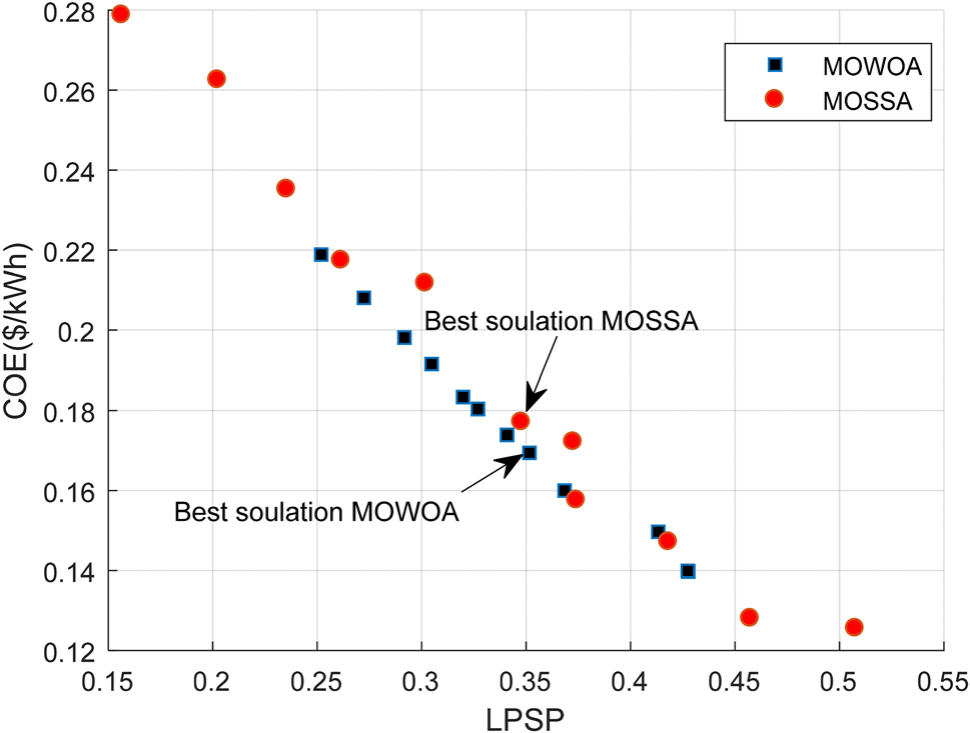
Comparison of Pareto fronts obtained for Case 3 using the two proposed approaches.

This design employs two optimization methods to optimize three components of the PV/WT/BES system in a standalone microgrid, resulting in the lowest total energy among the examined configurations. The Pareto front of the standalone microgrid system derived for each algorithm based on LPSP, COE, and RF functions is shown in Fig. 12, where the MOSSA algorithm consistently obtains the optimal solution. It has been demonstrated that the MOSSA method is more effective and efficient than other algorithms at producing better Pareto solutions. Consequently, the MOSSA approach can be effectively employed to optimize various microgrid configurations and to identify the most reliable and cost-effective design options. Based on Pareto fronts, both algorithms provide ten solutions, which are listed in Tables 9 and 10. These solutions have been arranged according to the LPSP for greater clarity. The MOSSA algorithm introduced the most suitable results for the case under study. In this configuration, the COE is 0.16496 $/kWh. The results obtained had met the predetermined constraints, LPSP is 35.882%, the PV system supplied 20.884 MW, and the days of autonomy for BESU were set to 1.7507. The COE varies between 0.12557$/kWh and 0.27883$/kWh, as provided in Table 9. With five WT required, 15.541 kW of power produced by the PV system, and 2.1592 autonomous days, Solution #1 has the lowest COE at 0.12557$/kWh. Solution # 10 met the highest COE at 0.27883$/kWh, resulting from ten WTs, the PV panels produce 36.103 kW of power, and the autonomous days equal 4.9864.

**Table 9:** Optimization Results for the MOSSA method under Case 3.

Solution	PV (kW)	BAD	WT	COE ($/kWh)	LPSP
1	15.471	2.3154	9	0.14965	0.41325
2	20.884	1.7507	9	0.16496	0.35882
3	20.884	2.0819	9	0.16942	0.35163
4	21.047	1.9702	10	0.17138	0.34526
5	23.27	1.7068	9	0.17531	0.33776
6	22.94	2.1401	9	0.1788	0.33053
7	24.182	2.2516	10	0.18888	0.30949
8	25.366	2.583	10	0.19812	0.29178
9	24.889	2.8581	10	0.19982	0.28834
10	26.224	2.9935	10	0.20807	0.27234

**Table 10:** Optimization Results for the MOWOA method under Case 3.

Solution	PV (kW)	BAD	WT	COE ($/kWh)	LPSP
1	15.541	2.1592	5	0.12557	0.50757
2	19.259	1.6017	5	0.13518	0.45042
3	23.689	1.1066	5	0.14719	0.41818
4	22.497	3.1081	5	0.17221	0.37261
5	21.536	4.9101	9	0.21172	0.3018
6	26.41	4.1195	8	0.21748	0.26151
7	29.319	4.7419	9	0.24259	0.21221
8	38.53	4.0211	7	0.26259	0.20233
9	42.423	4.3367	5	0.27304	0.20138
10	36.103	4.9864	10	0.27883	0.15643

C. **Case 4: PV/WT/BES/DG Design**.

**Fig. 13:**
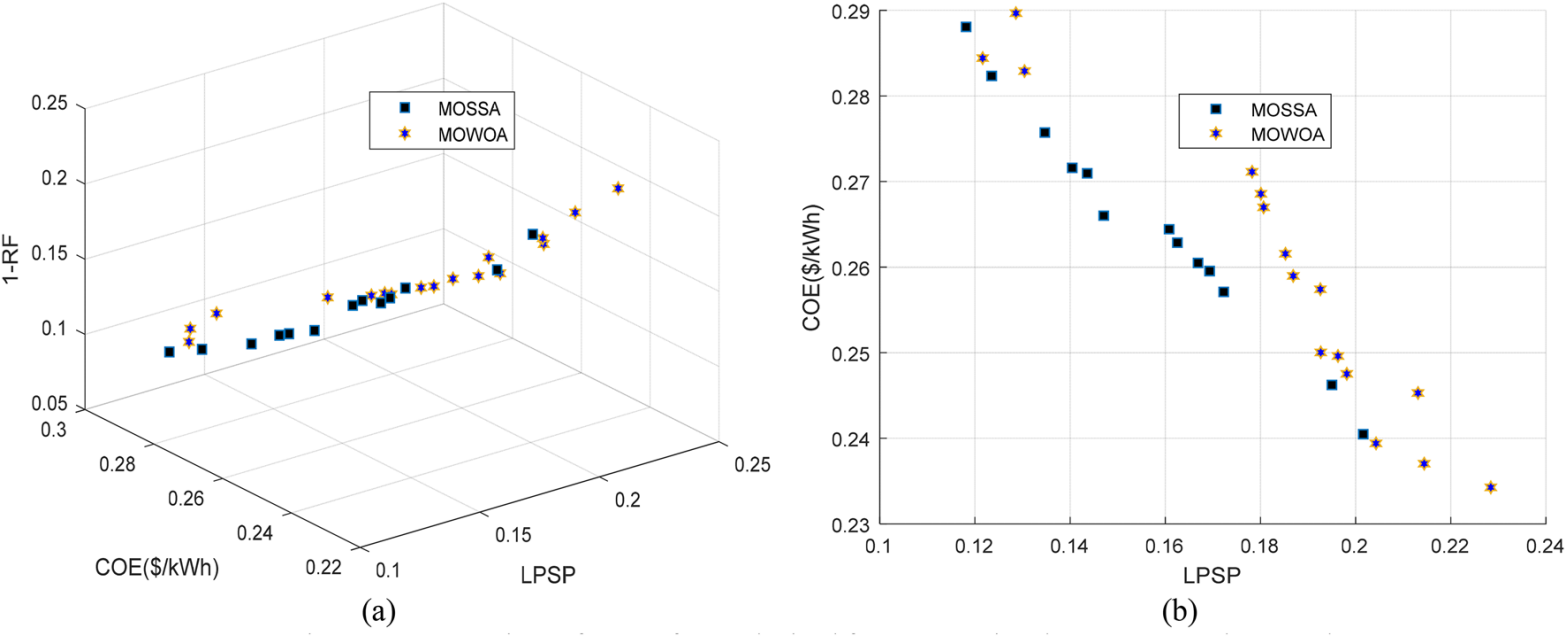
Comparison of Pareto fronts obtained for Case 4 using the two proposed approaches.

This design that produces the highest total energy is composed of four parts. The BES and DG provide backup power if other sources are insufficient, while the PV and WT provide renewable energy. The Pareto front of the standalone microgrid system derived for each algorithm based on LPSP, COE, and RF functions is shown in Fig. 13. The solutions of MOSSA dominate the MOWOA solutions, particularly in the low-loss and high-cost region, as seen in Fig. 13 (b). A detailed comparison of the performance of the MOSSA and MOWOA algorithms is presented in the following section.

Table 11 displays the ten chosen solutions from the Pareto fronts for the MOSSA method. As can be shown, option #1 has a higher LPSP value and lower COE and RE values than the other solutions (a COE of 0.2394 $/kWh, an LPSP of 20.428%, and an RF of 81.548%), the system requires seven WTs and one DG, the PV system generates 24 kW of power, and the BAD period is two days. Solution #10 has the lowest LPSP value, and the best COE and RE values (6.1989% LPSP, 0.46968 $/kWh, and 88.19% RF). In this solution, the PV system generates 41.572 kW of power, one DG and seven WTs will be required, and the autonomy period will be five days.

The MOWOA optimization results are given in Table 12. According to the outcomes, if the designer chooses option #1, the PV system generates 21.757 kW of power, the BAD period is 2.3623 days, and the system needs nine WTs and one DG. The COE, LPSP, and RF of this solution are 0.24047 $/kWh, 20.161%, and 81.292%, respectively. The COE and RE values are lower compared to other available solutions, while the LPSP is higher. If the designer chooses option #10, the PV system generates 36.791 kW of power, the BAD period is 3.8748 days, and the system requires eight WTs and two DGs. The COE, LPSP, and RF of this solution are 0.38952 $/kWh, 5.3391%, and 85.291%, respectively. The LPSP is lower than in the other solutions, while the COE and RE values are higher, illustrating the trade-off between system reliability and economic performance.

**Table 11:** Optimization Results for the MOSSA method under Case 4.

Solution	PV (kW)	BAD	WT	Diesel	COE($/kWh)	LPSP	RF
1	24	2	7	1	0.2394	0.20428	0.81548
2	27.98	1	8	1	0.24529	0.21312	0.8303
3	27.344	2.654	9	1	0.26128	0.16748	0.86578
4	33.776	4.3674	5	1	0.2829	0.13043	0.88076
5	34.612	3.3146	9	1	0.28968	0.12862	0.90665
6	37.612	5	6	1	0.30708	0.094028	0.91025
7	41.572	5	7	1	0.32663	0.078555	0.92861
8	44.566	5	7	1	0.34008	0.071921	0.93592
9	28.365	4.9689	10	2	0.37691	0.055514	0.83394
10	40.869	5	10	3	0.46968	0.0061989	0.8819

**Table 12:** Optimization Results for the MOWOA method under Case 4.

Solution	PV (kW)	BAD	WT	Diesel	COE($/kWh)	LPSP		RF
1	21.757	2.3623	9	1	0.24047	0.20161	0.81292	
2	25.619	1.7175	9	1	0.24624	0.195	0.84003	
3	25.632	3.0863	8	1	0.25922	0.16871	0.85454	
4	31.97	1.6444	9	1	0.27113	0.17824	0.87782	
5	30.493	3.9178	10	1	0.28233	0.12352	0.90071	
6	32.361	3.9145	9	1	0.28806	0.11813	0.90656	
7	29.921	3.6509	10	2	0.37446	0.062068	0.83372	
8	31.454	3.6509	10	2	0.37709	0.059927	0.84418	
9	33.408	3.6509	10	2	0.38208	0.056857	0.85489	
10	36.791	3.8748	8	2	0.38952	0.053391	0.85291

D. **A comparative analysis of the contributions of microgrid energy generation**.

**Fig. 14:**
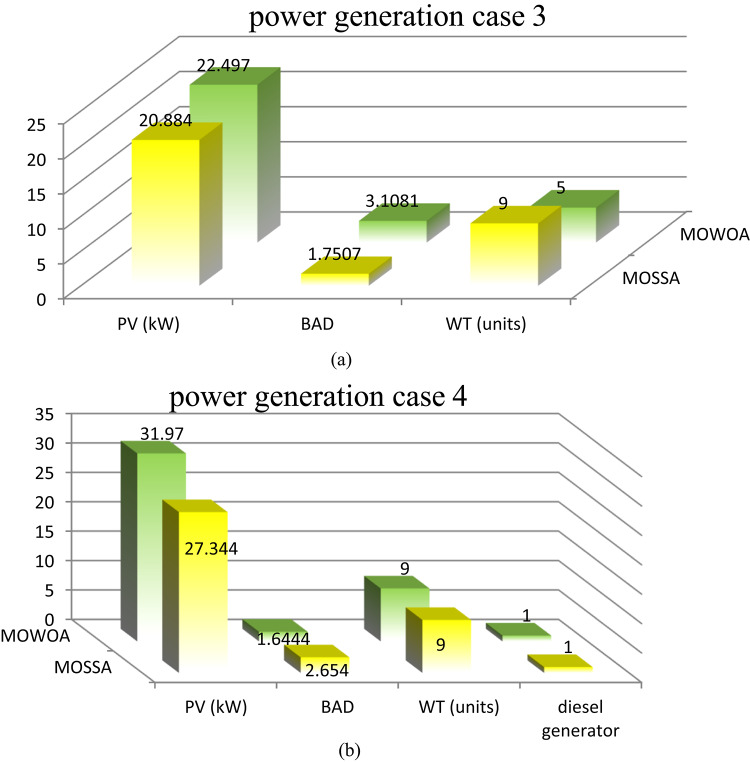
Comparison of the component sizes of the proposed architecture under different proposed methods.

Two different design configurations of microgrid components, PV/WT/BES/DG and PV/WT/BES, are examined. To solve the optimization problem examined in this research, the MOSSA method’s performance has been contrasted with that of the MOWOA algorithm. The energy contributions of various sources, such as PV system, WT, BES, and DG are contrasted in Fig. 14 (a, b) and Table 13. The results demonstrated that:

- PV/WT/BES/DG design comprises four components, and it provides the highest total energy. The renewable energy is supplied by PV and WT, while the DG and BES provide backup power when other sources fall short.
- With only three components, the PV/WT/BES design generates lower energy than the PV/WT/BES/DG architecture. In this architecture, the BES serves as the sole backup source when renewable energy sources are insufficient.
- The MOWOA approach contributes to a higher PV capacity than the MOSSA algorithm.
- This scenario has a greater range of RES sizes than that found in the previous scenario.
- **For the PV/WT/BESU architecture**.
- The MOSSA algorithm penetrates high WT (close to the maximum limitations of 10 WTs).
- The MOSSA algorithm created the lowest optimal objective function; the PV system’s yearly energy contribution is 26.224 kW, which appears in solution #10.
- Compared to the MOSSA, the MOWOA method obtained a higher number BES as well as a larger total PV capacity with lower average number of WT.
- The MOSSA algorithm penetrates high PV average size and BAD.
- Compared to the MOSSA, the MOWOA method obtained a higher number of WT and DG.
- **For the PV/WT/BESU/DG architecture**.
- The power contributed by PV units varies between 24 and 41.572 kW for the MOSSA approach and between 21.757 and 36.791 kW for the MOWOA method.
- WT units derived from the MOWOA close to the upper boundaries of^10^.
- According to the MOWOA technique, BADs vary between 1.6444 and 3.9178 days.
- The range of WT units taken from the MOSSA is six to ten units.
- The number of DG for MOWOA and MOSSA techniques is in the range of^1,2^ and^1–3^, respectively.

**Table 13:** Min, Max and average Limits Of Size of different Energy Resources in Scenario 2.

Component	limits	Case 3		Case 4	
		MOSSA	MOWOA	MOSSA	MOWOA
PV panel	Min	15.471	15.541	24	21.757
	Max	26.224	42.423	44.566	36.791
	Average	22.51	27.53	34.069	29.94
BAD	Min	1.7507	1.1066	1	1.6444
	Max	2.9935	4.9864	5	3.9178
	Average	2.26	3.5	3.38	3.14
WT	Min	9	5	7	8
	Max	10	10	10	10
	Average	9.5	6.8	7.8	9.2
DG	Min	--	--	1	1
	Max	--	--	3	2
	Average	--	--	1.3	1.4

E. **Comparative analysis of the LPSP**,** COE**,** and RF.**

**Fig. 15:**
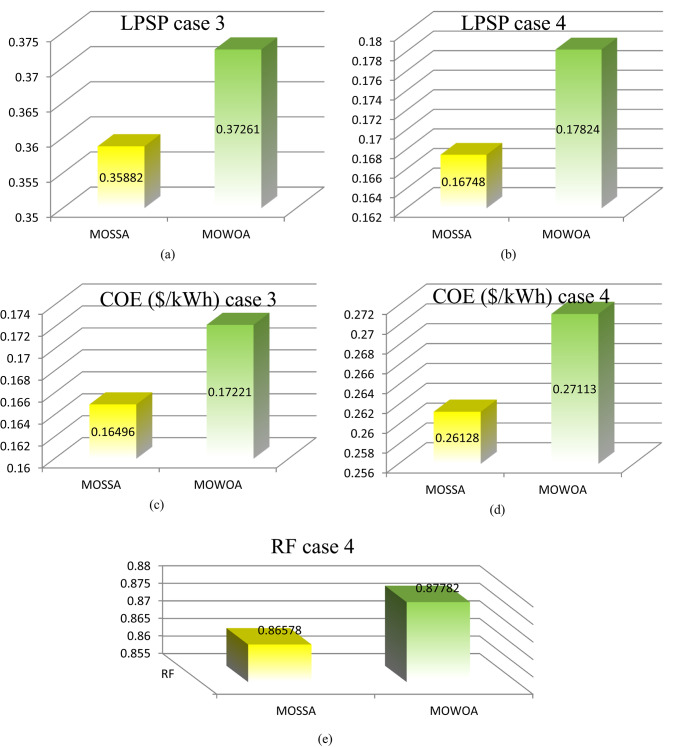
Comparison of LPSP, COE, and RF of the proposed architecture under different proposed methods.

Figure 15 (a, b, c, d, and e) displays the numerical fluctuations of the LPSP, COE, and RF indices in different models for various instances. As can be observed,

- Although the total amount of RES is higher than in the previous scenario, the cost of RES increased in this scenario.
- The PV/WT/BES/DG design produces more COE and less LPSP than the PV/WT/BES architecture due to its four components.
- The PV/WT/BES/DG design has an LPSP of less than 17%, while the PV/WT/BESU design has an LPSP of less than 50%, demonstrating that the limitations are maintained.
- According to the MOWOA algorithms, the REF performed well in all cases, surpassing 81.292%.
- **For the PV/WT/BESU/DG architecture**.
- MOSSA provided the best solution among all the optimal solutions, with a COE of 0.26128 $/kW h and an LPSP of 16.748%.
- The MOWOA algorithm produced more expensive results than MOSSA, with a COE of 0.27113 $/kW h and an LPSP of 17.824.
- **For the design of PV/WT/BES architecture**.
- According to the findings of the MOSSA approach, option #2 is the most suitable result for the case study. The COE of the system in this configuration is 0.16496 $/kWh, the BESU autonomy day is set at 1.7507, the LPSP is 35.882%, and the system receives 20.884 kW of power from PV.
- Based on the MOWOA methodology, the results show that Case # 4 is the most appropriate outcome for the case study. In this setup, the system’s COE is 0.17221 $/kWh. The constraints have been satisfied: the BESU autonomy day is set at 3.1081, the LPSP is 37,261%, and the system receives 22.497 kW of power from PV.
- Compared to the MOSSA, the MOWOA algorithms provided less costly and more loss solutions.

## Scenario 3: heavy loading condition

In this scenario, a microgrid system operates at normal load conditions, serving a community of 15 houses. The proposed multi-objective optimization methods are employed to simultaneously minimize the COE and the LPSP while maximizing the utilization of RES. This is achieved through the optimal allocation of a combination of RES (PV and WT), BES, and non-RES such as diesel generators.

### A. Case 5

B. **PV/WT/BES Design**.

**Fig. 16:**
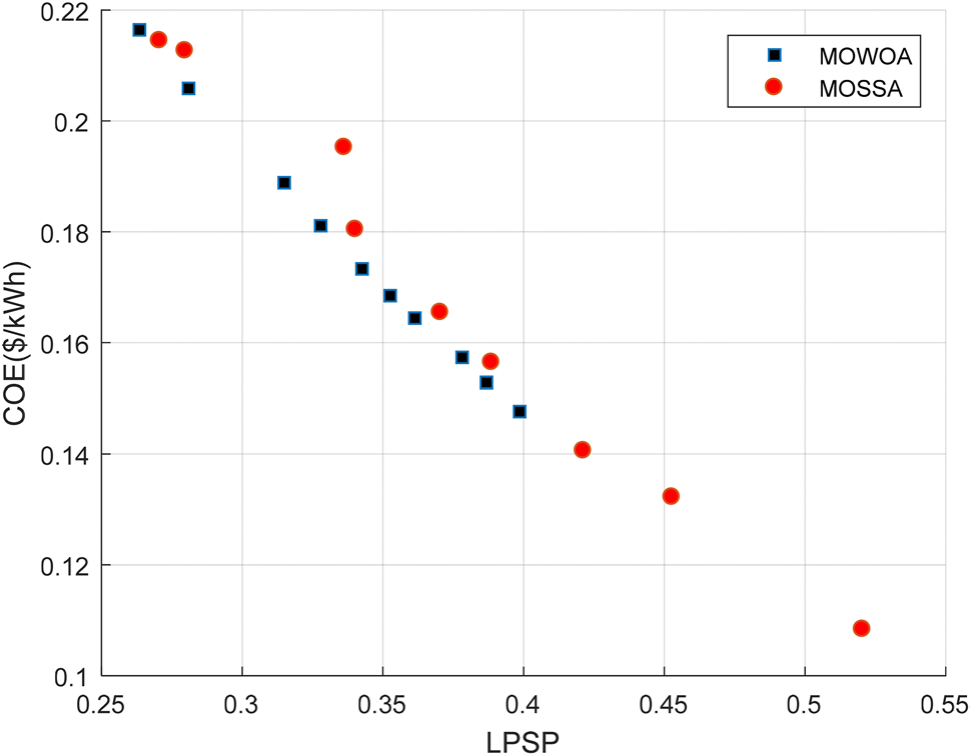
Comparison of the Pareto fronts obtained for Case 5 using the two proposed approaches.

In this case, the PV/WT/BES configuration contributes the least total energy, as it integrates only three components into the system. The Pareto front of the standalone microgrid system, obtained for each algorithm based on LPSP, COE, and RF objectives, is shown in Fig. 16. The results consistently indicate that the MOSSA algorithm provides the optimal solutions. It has been demonstrated that the MOSSA method is more effective and efficient than other algorithms at producing better Pareto solutions, particularly in the low COE and high LPSP portion.

Ten chosen solutions from the Pareto fronts for the two algorithms (MOSSA and MOWOA) are summarized in Tables 14 and 15, respectively. These solutions have been arranged according to the LPSP for greater clarity. The results based on the MOSSA method are displayed in Table 14. As shown, option #1 has a higher LPSP value and lower COE values than the other solutions, with a COE of 0.14765 $/kWh, an LPSP of 39.861%. In contrast, Solution #10 has the lowest LPSP value, and the best COE values, with a COE of 0.23086 $/kWh, 23.67% LPSP.

The MOWOA optimization results are presented in Table 15. According to the outcomes, if the designer chooses option #10, the PV system generates 34.811 kW of power, the BAD period is 3.9247 days, and the system requires eight WTs. The COE and LPSP of this solution are 0.18917 $/kWh, and 33.732%, respectively. In this case, the COE is higher than the other solutions, while the LPSP is comparatively lower, highlighting the trade-off between economic cost and system reliability.

**Table 14:** Optimization Results for the MOSSA method under Case 5.

Solution	PV (kW)	BAD	WT	COE ($/kWh)	LPSP
1	29.484	1.4476	10	0.14765	0.39861
2	28.734	1.7573	10	0.14991	0.39634
3	30.761	1.7573	10	0.15614	0.38052
4	31.353	1.7638	10	0.15735	0.37819
5	32.947	1.9644	10	0.16677	0.35652
6	34.684	2.4141	10	0.1782	0.33318
7	36.422	2.2652	10	0.18109	0.32792
8	40.085	3.5285	9	0.20587	0.28095
9	42.698	3.9648	8	0.21643	0.26347
10	45	4.5524	8	0.23086	0.2367

**Table 15:** Optimization Results for the MOWOA method under Case 5.

Solution	PV (kW)	BAD	WT	COE($/kWh)	LPSP
1	17.264	1	3	0.079633	0.66134
2	23.678	1	2	0.09491	0.60213
3	20.632	1.1734	7	0.10837	0.52043
4	27.767	1.8676	3	0.12473	0.51237
5	29.285	1.338	6	0.13223	0.45271
6	34.164	1.593	8	0.15646	0.38862
7	31.471	2.5684	9	0.16546	0.37043
8	37.202	2.3131	8	0.17628	0.34507
9	39.482	1.9626	8	0.18043	0.34025
10	34.811	3.9247	8	0.18917	0.33732

C. **Case 6: PV/WT/BES/DG Design**.

**Fig. 17:**
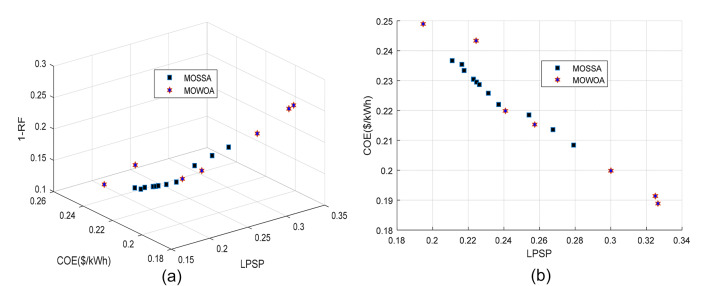
Comparison of the Pareto fronts obtained for CASE 6 using the two proposed approaches.

This configuration that produces the highest total energy is composed of four components. The BES and DG provide backup power if other sources are insufficient, while the PV and WT provide renewable energy. The Pareto front of the standalone microgrid system obtained for each algorithm based on LPSP, COE, and RF functions is illustrated in Fig. 17. The outcomes of the MOSSA and MOWOA algorithms are shown in the following section.

Table 16 displays the ten selected solutions based on the Pareto fronts obtained using the MOSSA method. According to the outcomes, solution #1 exhibits the highest LPSP and lowest COE and RE among all solutions, with a COE of 0.16673 $/kWh, an LPSP of 54.09%, and an RF of 16.871%. This configuration requires one WT and one DG, with the PV panels generating 16.049 kW of power, and the ADB period is one day. In contrast, Solution #10 achieves the lowest LPSP (7.4687%) and the highest energy contribution from PV panels (34.848 kW), with the BAD period is five days. This configuration requires ten WTs and three diesel generators.

The MOWOA optimization results are listed in Table 17. As shown, if the designer chooses option #1, the PV panels generate 38.328 kW of power, the BAD period is 2.8052 days, and the system requires ten WTs and one DG. The COE, LPSP, and RF of this solution are 0.23667 $/kWh, 21.099%, and 88.86%, respectively. The COE and RE have greater amounts than the other solutions that are available, while the LPSP has fewer values. If the designer chooses option #10, the PV panels generate 27.201 kW of power, the BAD period is 2.102 days, and ten WT and one DG are needed. The COE, LPSP, and RF of this solution are 0.20847 $/kW h, 27.914%, and 81.519%, respectively. In this case, the LPSP is higher than in the other solutions, while the COE and RF values are comparatively lower, illustrating the trade-off between system reliability and economic performance.

**Table 16:** Optimization Results for the MOSSA method under Case 6.

Solution	PV (kW)	BAD	WT	Diesel	COE ($/kWh)	LPSP	RF
1	16.049	1	1	1	0.16673	0.5409	0.16871
2	18	1	7	1	0.18087	0.38293	0.62037
3	17.631	1	7	1	0.18	0.38715	0.60995
4	25.049	1	7	1	0.18845	0.33627	0.72882
5	21	1.0304	10	1	0.18886	0.32649	0.74279
6	25.848	1	8	1	0.19136	0.32504	0.75066
7	32.712	1.6229	10	1	0.21529	0.25732	0.85301
8	35.011	2.4044	10	2	0.32651	0.15309	0.76971
9	39.07	3.9269	10	2	0.33504	0.11894	0.82109
10	34.848	5	10	3	0.4692	0.074687	0.72076

**Table 17:** Optimization Results for the MOWOA method under Case 6.

Solution	PV (kW)	BAD	WT	Diesel	COE ($/kWh)	LPSP		RF
1	38.328	2.8052	10	1	0.23667	0.21099	0.8886	
2	40.697	2.352	10	1	0.2385	0.21304	0.89445	
3	39.358	2.1133	10	1	0.23249	0.22332	0.88808	
4	38.796	2.1961	10	1	0.23151	0.22348	0.88592	
5	37.602	2.359	10	1	0.23044	0.22297	0.88353	
6	36.636	2.1341	10	1	0.22579	0.23125	0.87778	
7	34.725	2.3178	10	1	0.22258	0.23605	0.86896	
8	35.21	2.1133	10	1	0.222	0.23703	0.87165	
9	28.516	2.2835	10	1	0.21358	0.26755	0.8306	
10	27.201	2.102	10	1	0.20841	0.27914	0.81529

D. **A comparative analysis of the contribution of microgrid energy generation**.

**Fig. 18:**
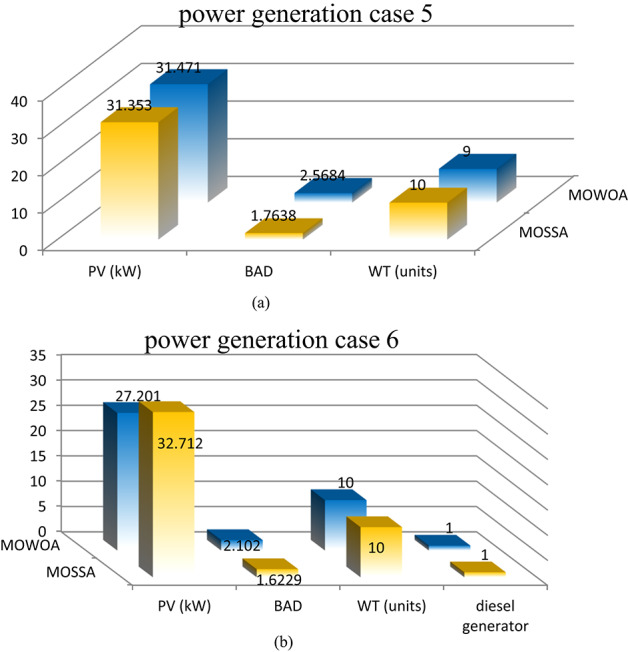
Comparison of the component sizes of the proposed architecture under different proposed methods.

An analysis was conducted to evaluate the energy supplied by different microgrid components for architectural combinations: PV/WT/BESU/DG and PV/WT/BES. To solve the optimization problem examined in this research, the MOSSA method’s performance was compared with that of the MOWOA algorithm. The energy contributions of various sources, such as PV panels, WT, BAD, and DG, are contrasted in Fig. 18 (a, b) and Table 18. The results demonstrated that:

- Compared to the PV/WT/BES/DG combination, the PV/WT/BES design produces less energy, since PV/WT/BES/DG combination uses four components, and the PV/WT/BES structure uses three components.
- The problem of MOWOA is assigning a large number and size of PV panels; the total capacity of PV obtained with the MOWOA algorithm is greater than that obtained from the MOSSA algorithm.
- This scenario has a greater range of RES sizes than that found in the previous scenario.
- The RF value obtained based on the MOWOA algorithm is better than the value obtained based on the MOSSA method.
- The optimal solution, which appears in solution #4 based on the MOSSA algorithm, has an annual contribution of 31.353 kW from the energy generated by the PV panels.
- For case 5, the MOSSA method obtained a highest average number WT and BES as well as a largest total PV capacity.
- **For the PV/WT/BESU/DG architecture**.
- Due to an increase in the load, the sizes of PV panels are increased to 40.697 kW based on the MOWOA algorithm.
- The number of diesel units ranged from [1 to 3] units based on the MOSSA algorithm.
- The MOSSA algorithm closes the diesel and WT unit numbers to one and ten, respectively.
- The range of RES sizes in this scenario is increased compared to the sizes in the two previous scenarios.
- Compared to the MOSSA, the MOWOA method obtained a higher number WT and BES as well as a larger total PV capacity with lower average number of DG.

**Table 18:** Min, Max and average Limits Of Size of different Energy Resources in Scenario 3.

Component	limits	Case 5		Case 6		
		MOSSA	MOWOA	MOSSA		MOWOA
PV panel	Min	28.734	17.264	16.049		27.201
	Max	45	39.482	39.07		40.697
	Average	35.31	26.798	26.521		35.7
BAD	Min	1.7573	1	1		2.102
	Max	4.5524	3.9247	5		2.8052
	Average	2.541	1.874	1.898		2.066
WT	Min	8	3	1		10
	Max	10	8	10		10
	Average	9.5	6.2	7.3		10
DG	Min	--	--	1		1
	Max	--	--	3		1
	Average	--	--	1.4		1

E. **Comparative analysis of the LPSP**,** COE**,** and RF.**

**Fig. 19:**
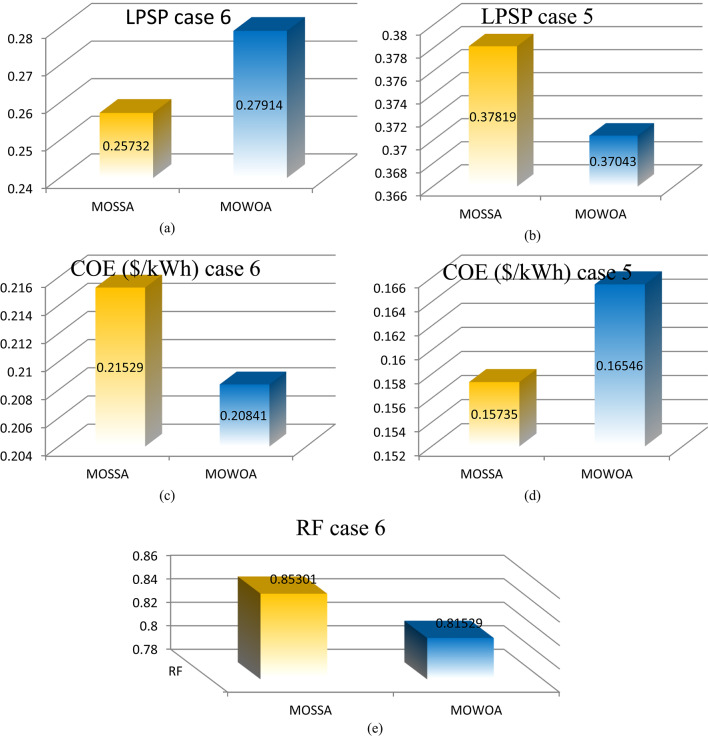
Comparison of LPSP, COE, and RF of the proposed architecture under different proposed methods.

Figure 19 (a, b, c, d, and e) displays the numerical fluctuations of the LPSP, COE, and RF indices in different models for various instances. As can be observed,

- Although the overall capacity of RES is higher than in the two previous scenarios, the cost of RES increased under this scenario.
- The PV/WT/BESU/DG design produces more COE and less LPSP than the PV/WT/BESU architecture due to it having four components.
- The PV/WT/BESU/DG design has an LPSP of less than 38%, while the PV/WT/BESU design has an LPSP of less than 66%.
- **For the PV/WT/BESU/DG architecture**.
- The cost of the integrating units, calculated using the MOSSA approach, falls between 0.16673 and 0.4692 $/kWh.
- According to the MOWOA approach, the cost of the integrating units falls between 0.20841 and 0.2366 $/kWh.
- The MOWOA algorithm’s LPSP value falls between 21.099 and 27.914%.
- The LPSP values for the MOSSA technique varied from 7.4687 to 52.409%.
- MOWOA outperformed the MOSSA algorithm in terms of RE results.
- In all cases, the obtained results according to MOWOA algorithms introduce a better REF factor, which exceeds 89.445%.
- **For the design of PV/WT/BESU architecture**.
- According to the findings of the MOSSA approach, the units’ costs fall between 0.079633 and $0.18917 $/kWh.
- The MOWOA method estimates the cost of the connected units, which falls between 0.14765 and 0.23086 $/kWh.
- The MOWOA algorithm gives an LPSP value between 23.67 and 39.861%.
- The range of LPSP values for the MOSSA technique was 33.732 to 66.14%.
- Compared to the MOSSA, the MOWOA algorithms provided less costly solutions.

### Comparative analysis of the contribution of microgrid power generation depending on various load levels

**Fig. 20:**
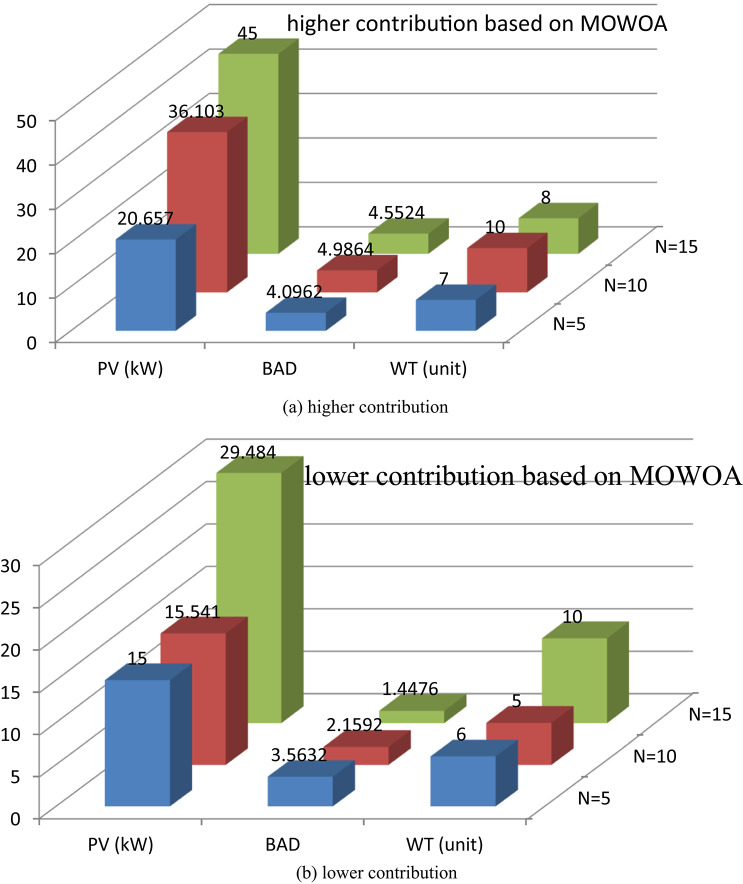
Generated power via each component (PV, WT, and AD) according to the number of houses.

Figure 20 (a) and (b) show the generated power via each component (PV, WT, and BES) resulting from the methodology used (MOWOA method) according to various load levels. As the load level increases, so do the contribution limits of all power-producing sources. The maximum capacities of the hybrid renewable-based microgrid system are as follows:

- **Scenario 1 (N = 5)**: PV = 20.657 kW, WT = 7 units, BAD = 4.0962 days.
- **Scenario 2 (N = 10)**: PV = 36.103 kW, WT = 10 units, BAD = 4.9864 days.
- **Scenario 3 (N= 15)**: PV = 45 kW, WT = 8 units, BAD = 4.5524 days.

The minimum capacities of the hybrid renewable-based microgrid system for the three scenarios are as follows:

- **Scenario 1 (N = 5)**: PV = 15 kW, WT = 6 units, BAD = 3.5632 days.
- **Scenario 2 (N = 10)**: PV = 15.541 kW, WT = 5 units, BAD = 2.1592 days.
- **Scenario 3 (N = 15)**: PV = 29.484 kW, WT = 10 units, BAD = 1.4476 days.

For Fig. 20(a), due to an increase in the load,

- The contribution of PV rises from 20.657 kW in Scenario 1 to 36.103 kW in Scenario 2, and further to 45 kW in Scenario 3.
- The number of WTs increases from 7 units for 5 houses to 10 units for 10 houses. Unlike scenario 3, the number of WT reduced to 8 units due to high contribution of PV.
- The BADs increase from 4.0962 in scenario 1 to 4.9864 in scenario 2. In contrast, in Scenario 3, when the higher PV contribution reduces the autonomy period to 4.5524.

The obtained techno-economic results are consistent with trends reported in recent hybrid renewable microgrid optimization studies conducted in regions with comparable solar and wind resource availability. The achieved RF values demonstrate the feasibility of high renewable penetration while maintaining acceptable reliability levels. In comparison with similar microgrid planning studies reported in the literature, the optimized COE values fall within the competitive range typically observed for hybrid PV/WT/BESS/DG systems under favorable climatic conditions. Furthermore, the observed trade-off between COE minimization and reliability enhancement aligns with previously reported findings, in which increased renewable penetration and storage capacity generally improve reliability at the expense of higher initial capital investment. The convergence behavior and Pareto front distribution observed for the proposed multi-objective framework also reflect patterns reported in other metaheuristic-based microgrid sizing studies, where adaptive exploration–exploitation balance contributes to stable multi-objective trade-off identification. Overall, the results confirm that the proposed optimization strategy produces technically and economically viable configurations comparable to those reported in existing literature, while offering structured multi-objective decision-making capability.

### Performance characteristics of MOSSA and MOWOA

The foundational studies of the SSA and WOA demonstrated competitive performance compared with conventional metaheuristic algorithms such as GA, PSO, and DE in various benchmark optimization problems. Building upon these established properties, the present study evaluates their multi-objective variants, MOSSA and MOWOA, within the context of hybrid microgrid sizing. Based on the obtained simulation results, MOSSA exhibits strong exploration capability due to its leader–follower chain structure, which contributes to improved Pareto front diversity and reduced risk of premature convergence. This behavior enables broader trade-off coverage among COE, LPSP, and renewable fraction objectives. Conversely, MOWOA demonstrates stable and smooth convergence patterns, particularly in later optimization stages, owing to its encircling and spiral updating mechanisms that enhance local exploitation efficiency. Nevertheless, both algorithms remain sensitive to parameter selection and population size configuration, which may influence convergence speed and computational cost. Additionally, while their global search capability has been validated in prior benchmark studies, performance in large-scale or highly constrained real-world energy systems may require further investigation.

From a computational perspective, both MOSSA and MOWOA were executed under identical population size and iteration settings to ensure fair comparison. Since both are population-based metaheuristic algorithms, their computational complexity is primarily proportional to the number of agents and objective function evaluations per iteration. MOSSA involves leader–follower positional updates with relatively straightforward mathematical operations, resulting in moderate per-iteration computational overhead. In contrast, MOWOA requires additional encircling and spiral position update calculations, which may slightly increase computational effort per iteration. However, the overall runtime in this study is predominantly influenced by the evaluation of the multi-objective fitness functions rather than the internal update equations. For the microgrid sizing problem considered, both algorithms demonstrated acceptable computational performance suitable for planning-scale applications.

### Research limitations and future directions

Despite the comprehensive techno-economic and reliability assessment conducted in this study, several limitations and practical implementation challenges should be acknowledged. First, the residential load demand is modeled deterministically under three predefined scaling scenarios without incorporating stochastic load uncertainty, long-term demand growth forecasting, or demand-side management strategies. In real-world applications, load profiles are inherently dynamic and may exhibit seasonal, behavioral, and socio-economic variations, which could influence optimal system sizing and operational performance. Incorporating probabilistic load modeling and adaptive scheduling mechanisms would enhance the robustness of the proposed framework. Second, the optimization framework is validated using meteorological data from Yanbu, Saudi Arabia. Although the proposed methodology is generic and can be applied to other geographical locations, system performance may vary under different climatic conditions, renewable resource intermittency levels, and seasonal variability patterns. Therefore, multi-location assessment and climate sensitivity analysis would provide a broader evaluation of the model’s scalability and adaptability. Third, the adopted energy management strategy (EMS) is rule-based and does not incorporate predictive, adaptive, or artificial intelligence-driven dispatch techniques. While the rule-based EMS ensures operational simplicity and computational efficiency, more advanced predictive or model-based control approaches may further improve system flexibility, reliability, and economic performance under uncertain operating conditions.

In addition, economic parameters such as capital costs, fuel prices, discount rates, and operation and maintenance expenses are assumed to be constant throughout the project lifetime. However, real-world market fluctuations and policy changes may significantly influence long-term economic feasibility. Conducting comprehensive sensitivity and uncertainty analysis would strengthen the financial robustness of the proposed system design. Practical deployment challenges may also arise, including land availability for photovoltaic and wind turbine installations, regulatory approvals, grid interconnection requirements, and maintenance logistics in remote or coastal regions. Furthermore, the findings of this study are derived from simulation-based optimization without experimental or hardware-in-the-loop validation. Future research will address these limitations through uncertainty-aware modeling, advanced EMS integration, multi-regional validation, economic sensitivity assessment, and real-time experimental implementation to further enhance the practical applicability of the proposed framework.

## Conclusion

This study proposed two optimization methods for determining the optimal sizing of a standalone microgrid system powered by combined energy sources, including solar PV panels, wind turbines (WT), battery energy storage systems (BES), and diesel generators (DG). The system was analyzed to meet the energy demand of residential communities in Yanbu, Saudi Arabia, considering varying numbers of households, with the objectives of maximizing renewable energy utilization while minimizing cost of energy (COE) and loss of power supply probability (LPSP).

The key findings and contributions of this study are summarized as follows:

1. Hybrid Microgrid Design: A multi-objective optimization framework was developed for standalone hybrid microgrid systems (HMS) integrating solar PV panels, wind turbines (WT), battery energy storage systems (BES), and diesel generators (DG) to supply residential communities in Yanbu, Saudi Arabia.
2. Scenarios and Configurations: System performance was evaluated for three residential scenarios (5, 10, and 15 houses) under two configurations: PV/WT/BES and PV/WT/BES/DG. This demonstrates the framework’s flexibility and scalability.
3. Optimization Methods: Two multi-objective optimization algorithms, MOSSA and MOWOA, were applied. MOSSA provided broader Pareto front coverage and higher renewable penetration, while MOWOA achieved competitive cost-effectiveness in certain scenarios.
4. Performance Highlights:

- The PV/WT/BES/DG configuration offered higher reliability (lower LPSP) and better renewable utilization compared to the PV/WT/BES system.
- Increasing the number of households enhanced the renewable fraction (RF) but slightly increased LPSP.
- The framework successfully balanced technical performance (LPSP) and economic metrics (COE) across all cases.

5. Design Implications: The results provide practical guidance for HMS designers and engineers to select optimal system configurations and component sizing that achieve a balance between reliability, renewable energy utilization, and economic performance.
6. Future Work:

- Incorporate stochastic load modeling and uncertainty-aware optimization to account for demand variability and renewable intermittency.
- Incorporate additional renewable sources, such as biomass or concentrated solar power, to further diversify the energy supply.
- Implement AI-based predictive energy management for dynamic load and generation conditions.
- Explore grid-connected operation, real-time pricing, and demand response strategies to enhance system efficiency and sustainability.

## Data Availability

All data generated or analyzed during this study are included in this article.

## List of symbols

AC: Alternating Current
*BAD*: The battery autonomy days
a: linearly reduced from 2 to 0.
CPV: The investment costs of the PV
CWT: The investment costs of the WT
CBatt: The investment costs of the AD
Cinv: The investment costs of the inverter
Cr Batt: The replacement costs of the BESU
Cr inv: The replacement costs of the inverter
COE: The cost of electricity
CRF: The capital recovery factor
DC: Direct Current
DOD: The depth of discharge (80%)
ES: energy storage
$$E_{L}$$: The load
fr: The annual inflation rate
$\:{\mathrm{F}}_{DG}\left(\mathrm{t}\right)$: The generator fuel consumption (L/hr)
G(t): The solar radiation (W̸̸̸$\:\:{\mathrm{m}}^{2})$;
G_ref_: 1 kW $$\:/{\mathrm{m}}^{2}$$
HMS: Hybrid Microgrid System
I: The real interest rate (%)
ir: Nominal interest rate
kt: Constant, -3.7$$\times10^{-3}$$ (1/C)
LPSP: Loss of power supply probability
MOSSA: Salp Swarm Algorithm
MOWOA: Whale Optimization Algorithm
Npv: Numbers of PV
Nwt: Numbers of WT
Nbatt: Numbers of BT
n: The lifetime of the HRES project (years)
O&M: Operation and Maintenance
$\:{P}_{DG}\left(t\right)$: The generated power (kW)
P_r_: The capacity of the generator (kW)
$\upbeta$: The fuel intercept coefficient
$\:{\mathrm{P}}_{{\mathrm{N}}_{-\mathrm{P}\mathrm{V}}}$: The PV rated power at standard test conditions (STC)
Ppv: The power output of the PV panels
Pwt: The power output of the WT units
Pbatt: The power outputs of the battery
$$\:{P}_{\begin{array}{c}rated\end{array}}$$: The rated power of the WT
P: A random number in [0, 1].
PF: Pareto Front
$\:{\mathrm{P}}_{{\mathrm{N}}_{-\mathrm{P}\mathrm{V}}}$: The PV rated power at standard test conditions (STC)
RES: Renewable Energy Source
r: The random vector in [0, 1]
TNPC: The total NPC
T_air_: The temperatures (°C) of the ambient air at the reference conditions
T_air_: The temperatures (°C) of the cell at the reference conditions
T_nom_: The nominal cell operating temperatures
t: The time
$v_{1}$: The speed at a reference height,$h_{1}$
$v_{2}$: The speed at a hub height, $h_{2}$
$v_{0}$: The initial speed
V: The current time step wind speed
$V_{rated}$: The nominal wind speed$V_{rated}$
$V_{cut-out}$: The cut-out wind speed
$V_{cut-in}$: The cut-in wind speed
WT: Wind Turbine
$x_{j}^{i}$: he position vector of the i th follower salp in the j th dimension
$\overrightarrow{{\rm X}^*}$: The position vector of the best solution obtained
$\overrightarrow{{\rm X}}$: The position vector
$\alpha$: The coefficient of friction
$\eta_{inv}$: The inverter efficiency (95%)
$\upeta_{\rm overall}$: The overall efficiency
$\upeta_{\rm brake\:thermal}$: The brake thermal efficienc
$\upeta_{\rm rl}$: The reference efficiencies
$\upeta_{\rm pc}$: The power conditioning module efficiencies
${\rm T}_{\rm amb}$: The ambient temperature
${\rm T_{ref}}$: The temperature of th PV cell at STC ($25^{\circ}{\rm C}$ )
$\upeta_{b}$: The battery efficiency (85%)
